# Sex‐Stratified Associations of DXA‐Derived Adiposity, Regional Fat Distribution and Muscle Power With Incident Hospitalised Cardiovascular Disease in Older Adults: A Prospective Cohort Study

**DOI:** 10.1111/dom.71360

**Published:** 2026-09-14

**Authors:** Ting Zhang, Yuan Gao, Yafei Wu, Jason Leung, Timothy Kwok

**Affiliations:** ^1^ Department of Medicine and Therapeutics Faculty of Medicine, The Chinese University of Hong Kong Hong Kong China; ^2^ Department of Geriatrics Renji Hospital, Shanghai Jiao Tong University School of Medicine Shanghai China; ^3^ Shanghai Jiao Tong University School of Medicine Shanghai China; ^4^ Jockey Club Centre for Osteoporosis Care and Control, The Chinese University of Hong Kong Hong Kong China

**Keywords:** adiposity, cardiovascular disease, muscle power, older adults, regional fat distribution, sex differences

## Abstract

**Aims:**

To investigate the sex‐stratified associations of DXA‐derived adiposity, regional fat distribution and muscle power with incident hospitalised cardiovascular disease (CVD) in older Chinese adults.

**Materials and Methods:**

This prospective cohort included 3168 community‐dwelling older adults (1548 men and 1620 women; median age 72.0 years). DXA assessed body composition and regional fat, and the sit‐to‐stand (STS) test assessed muscle power. Incident hospitalised CVD was ascertained through hospital records. We used sex‐stratified multivariable Cox models as primary analysis, with Fine–Gray models as sensitivity analyses.

**Results:**

During a median follow‐up of 5.7 years, 330 participants developed CVD (199 men, 131 women). In fully adjusted models, higher adiposity (body fat percentage, fat mass index and waist circumference) was significantly associated with increased CVD risk in men (HRs per 1‐SD: 1.24–1.25). Similarly, trunk fat measures, including trunk FMR and trunk FMI, were significantly associated with CVD risk in men, with sex interactions retaining significance after multiple‐comparison correction (both *q* interaction < 0.05). By contrast, no such associations were observed in women. Higher muscle power was associated with lower CVD risk in women only (HRs per 1‐SD: 0.77–0.79). The leg‐to‐total fat ratio was inversely associated with cardiometabolic risk factors in both sexes (all *p* < 0.05). Results remained robust in sensitivity analyses.

**Conclusions:**

In this cohort of community‐dwelling older adults, central adiposity was associated with higher CVD risk in men, while greater muscle power was associated with lower risk in women. These exploratory findings highlight the importance of sex‐stratified cardiometabolic risk assessment and warrant future validation.

## 1 Introduction

Cardiovascular diseases (CVDs) remain the leading cause of morbidity and mortality worldwide [1], with their burden significantly affecting older adults. Globally, CVD cases nearly doubled to 523 million by 2019, underscoring its growing healthcare costs [1]. The global demographic shift towards an aging population has intensified the need for effective primary prevention strategies targeting modifiable risk factors. Aging is associated with profound changes in body composition, including increased adiposity [2], redistribution of fat to central depots [3] and progressive loss of muscle mass and function [4].

Previous studies examining body composition and muscle function in relation to CVD risk have yielded inconsistent findings. Obesity, traditionally assessed by body mass index (BMI), has been linked to increased CVD risk; however, BMI alone cannot distinguish fat from lean mass (LM) nor capture fat distribution [5]. Bioelectrical impedance analysis (BIA) has been widely used in large‐scale studies [6, 7], but unlike the reference‐standard dual‐energy x‐ray absorptiometry (DXA), BIA cannot precisely quantify regional fat distribution [8].

Using DXA, some studies have yielded important but heterogeneous findings. Fukuda et al. showed that visceral‐pattern obesity combined with low muscle mass predicted CVD in diabetic patients [9] and Chen et al. demonstrated that inflammation mediated 23%–38% of the fat‐CVD association [10]. Comparing fat versus muscle, Bell et al. reported that fat gain was more strongly linked to adverse cardiometabolic traits than muscle gain in young adults [11]. By contrast, higher leg‐to‐trunk fat ratio has been linked to lower mortality, supporting a protective role of peripheral fat [12]. Srikanthan et al. also reported that in adults aged ≥ 20 years, high fat mass (FM) significantly reduced CVD mortality in women, independent of muscle mass, whereas in men, risk reduction was confined to the high muscle‐low fat phenotype [13]. Muscle strength has emerged as a robust predictor of CVD and all‐cause mortality in older populations [14, 15]. Similarly, muscle power is more closely related to physical function than muscle strength alone [16, 17]. However, most existing studies on muscle power have relied on specialised laboratory‐based equipment, limiting their applicability in large‐scale or community settings [17, 18]. Recently, the sit‐to‐stand (STS) muscle power test has been validated as a simple, inexpensive and portable alternative for assessing muscle power in older adults [16].

Despite these advances, several limitations remain. First, while DXA‐based studies have examined overall adiposity or specific fat depots separately, few have comprehensively evaluated the simultaneous associations of DXA‐derived overall adiposity, regional fat distribution and lower‐limb muscle power with incident CVD within the same cohort. Second, the role of peripheral fat distribution, particularly leg fat, remains controversial. Some studies suggested a protective role of leg fat (the ‘gluteofemoral paradox’) [19], whereas others reported null or even adverse associations [20]. Third, given that men and women exhibit distinct patterns of fat deposition (android vs. gynoid) [21] and differ in muscle mass, with aging‐related loss being most pronounced in the lower body [22], further sex‐stratified analyses of CVD risk in older adults are warranted. Fourth, while ethnic differences in body fat distribution [23], data from Asian cohorts remain limited. Finally, the independent contribution of STS‐derived muscle power is less clear.

Consequently, to our knowledge, no previous study has simultaneously evaluated the sex‐stratified associations of DXA‐derived body composition (overall adiposity, LM and regional fat), upper‐limb muscle strength and lower‐limb muscle power with incident CVD within the same community‐dwelling older Asian cohort. We hypothesised that (1) central adiposity would be positively associated with incident CVD in men and (2) STS‐derived muscle power would be inversely associated with incident CVD in women. To test these hypotheses, we investigated these associations in a large prospective cohort of older Chinese adults, with secondary exploratory analyses examining regional fat distribution and its correlations with metabolic risk factors.

## 2 Materials and Methods

### 2.1 Study Participants

This prospective analysis utilised data from the Mr. OS and Ms. OS (Hong Kong) cohort, a community‐based longitudinal study focused on older Chinese adults [24]. Recruitment was conducted between August 2001 and March 2003 through health talks and recruitment notices, targeting community‐dwelling individuals aged 65 years or older. A total of 4000 participants (2000 men and 2000 women) were initially enrolled, with stratified sampling ensuring balanced representation across three age groups: 65–69, 70–74 and ≥ 75 years. A participant flow diagram was provided in Figure S1. At baseline, participants with self‐reported prevalent CVD were excluded (*n* = 832), leaving an analytic cohort of 3168 individuals (1548 men and 1620 women). Follow‐up was complete for all participants through linkage to the Clinical Management System (CMS) database and Hong Kong death registry. To assess potential selection bias, we compared the baseline characteristics of participants excluded due to prevalent CVD with those retained in the analytic cohort (Table S1). The study was carried out in accordance with the Declaration of Helsinki and was approved by the Clinical Research Ethics Committee of The Chinese University of Hong Kong. All participants provided informed consent prior to enrollment.

### 2.2 Body Composition

At baseline, body composition, including FM and LM, was measured by trained technicians using DXA with a Hologic QDR 4500 W device (Waltham, MA, USA). Daily quality assurance and calibration were performed at least three times per week using a Hologic body composition step phantom. The maximum coefficients of variation were 1.47% for FM and 0.84% for LM. Appendicular lean mass (ALM) was calculated as the sum of LM in all four limbs. Waist circumference (WC) was measured at the midpoint between the lowest rib and the iliac crest. ALM was adjusted for body size to derive height‐adjusted ALM (ALM/ht^2^), weight‐adjusted ALM (ALM/weight) and BMI‐adjusted ALM (ALM/BMI).

Fat mass index (FMI) was calculated as total body FM divided by height squared (kg/m^2^), with regional FMI values derived for the trunk, arms and legs. The fat‐to‐muscle mass ratio (FMR) was defined as regional FM divided by regional LM for the trunk, arms and legs. Regional‐to‐total body fat ratios (trunk‐to‐total, arm‐to‐total and leg‐to‐total) were calculated by dividing regional FM by total body FM.

### 2.3 Muscle Strength and Gait Speed

Handgrip strength was measured using a calibrated JAMAR dynamometer (Hand Dynamometer 5030JO, Sammons Preston, Bolingbrook, IL, USA). For each hand, participants performed two trials and the maximum reading was recorded. Gait speed was calculated from the time taken to walk a 6‐m course at usual pace; the test was performed twice and the faster trial was used for analysis.

### 2.4 Muscle Power

Muscle power was assessed by the five‐repetition STS test [16, 25]. Participants were instructed to rise from a standardised chair (seat height 0.44 m) to a fully standing position and then return to the seated position, performing five consecutive cycles as quickly as possible, with arms crossed over the chest. The time required to complete the five cycles was recorded using a stopwatch. The STS test was completed by 3135 of 3168 participants (98.96%); the remaining 33 (1.04%) were unable or unwilling to complete it and were excluded from STS‐related analyses. Absolute STS muscle power (W) was computed using a validated equation following Alcazar et al. [16]. Allometric muscle power (W/m^2^) was derived by normalising absolute power to height squared and relative muscle power (W/kg) was obtained by dividing absolute power by body mass [25].

$$\begin{array}{c} \mathrm{STS}\;\text{power}\;\left(W\right)\\ =\frac{\text{Body mass}\;\left(\mathrm{kg}\right)\times 0.9\times 9.81\times \left[\text{Height}\;\left(m\right)\times 0.5-\text{Chair height}\;\left(m\right)\right]}{\text{Five}\;\mathrm{STS}\;\text{time}\;\left(s\right)\times 0.1} \end{array}$$

### 2.5 Data on Incident CVD

Incident CVD was defined as newly identified cases occurring during follow‐up [26], comprising ischemic heart disease (IHD; including myocardial infarction, angina and acute coronary syndrome), heart failure, ischemic and haemorrhagic stroke and transient ischemic attack. We used ICD‐9‐CM codes to define specific CVD subtypes: IHD (410–414), heart failure (428, 429.4, 398.91, 402.11, 639.8, 668.1 and 997.1), stroke (431, 433, 434 and 432.9) and transient ischemic attack (435). CVD events were identified using the discharge diagnosis from the CMS database of the Hong Kong Hospital Authority (HA), which covers over 95% of all hospital admissions in Hong Kong [27]. Incident CVD was ascertained from baseline to September 2008. Event rates by subtype and sex were presented in Table S2.

All‐cause and cause‐specific mortality were derived from the Hong Kong Government Death Registry, with deaths coded according to the ICD‐10. CVD‐related mortality was defined as the death record under I00‐I99 codes. CVD deaths without a prior hospital‐recorded CVD in the HA database were included as incident CVD events only in sensitivity analyses. Follow‐up duration was calculated from the baseline assessment to the date of the first CVD event, date of death or the censoring date, whichever occurred first. Non‐CVD deaths were treated as censoring events in the primary Cox analyses and treated as competing risks in Fine–Gray sensitivity models.

### 2.6 Covariates

Information on potential confounders was collected using a standardised questionnaire. Demographic variables included age, sex and educational attainment (no formal education, primary or below and secondary or above). Lifestyle factors included smoking status (never, former or current), alcohol consumption, physical activity and dietary energy intake. Medical history (diabetes, hypertension, cancer history) and medication use (e.g., lipid‐lowering agents) were also documented. Physical activity was evaluated using the physical activity scale of the elderly (PASE), which has been validated for the Chinese population in Hong Kong [28]. Dietary intake was evaluated using a food frequency questionnaire (FFQ) validated in the Hong Kong Chinese population [29]. Missing values for energy intake (*n* = 3, 0.09%) and alcohol drinking (*n* = 1, 0.03%) were imputed using the sex‐specific median and modal value, respectively (Table S3).

### 2.7 Statistical Analysis

Baseline characteristics were summarised using mean (standard deviation, SD) or median (interquartile range, IQR) for continuous variables and frequency (percentage) for categorical variables. Between‐group comparisons by sex were performed using independent‐samples *t*‐test, Mann–Whitney *U* test or *χ* ^2^ test, as appropriate.

To evaluate the associations of body composition, muscle strength and muscle power (per 1‐SD increment) with incident CVD, multivariable Cox proportional hazards regression models were used as the primary analysis, with results presented as hazard ratios (HRs) with 95% confidence intervals (CIs). Model 1 was adjusted for age in the sex‐stratified analyses (and additionally adjusted for sex in the total population). Model 2 was additionally accounted for education level, smoking status, alcohol drinking, physical activity, energy intake, diabetes, hypertension, cancer history and lipid‐lowering medication use. Sex interactions were tested using likelihood ratio tests with a sex × exposure term; Benjamini–Hochberg FDR correction was applied to interaction *p* values from Model 1 and Model 2 separately, with raw *p* values and FDR‐adjusted *q* values reported (*q* < 0.05 considered significant).

Sensitivity analyses comprised: (1) Fine–Gray sub‐distribution hazard models treating non‐CVD death as a competing risk; (2) expanded outcome definition including CVD deaths without prior hospitalisation; (3) complete‐case analysis (*n* = 3130); (4) mutually adjusted models using FMI for ALM/weight and ALM/height^2^ for FMR and fat ratios to address mathematical coupling in ratio‐based exposures; (5) STS power mutually adjusted for FMI and ALM/height^2^; (6) a 2‐year lag analysis excluding early CVD events to reduce potential bias from subclinical disease or frailty; (7) additional adjustment for measured blood pressure, creatinine and homocysteine in the available subset and (8) separate examination of major CVD components (CHD, stroke and heart failure), with participants having corresponding baseline conditions excluded for each component.

Restricted cubic spline (RCS) regression with three knots (10th, 50th, 90th percentiles) was used to characterise dose–response relationships. The proportional‐hazards assumption was assessed using Schoenfeld residuals. Missing data for STS‐derived measures and waist were handled by complete‐case analysis. Sex‐stratified SD values for per‐1‐SD HR calculations were presented in Table S4 and statistical software details in Table S5. Effective sample sizes and event counts were indicated in table headers and footnotes. All tests were two‐sided, with *p*‐value < 0.05 considered statistically significant. All analyses were performed using R (version 4.4.3) and IBM SPSS (version 30.0).

## 3 Results

### 3.1 Baseline Characteristics of Study Participants

As shown in Table 1, the analytic cohort comprised 3168 participants (1548 men and 1620 women), with a median age of 72.0 years. Participants excluded due to prevalent CVD (*n* = 832) had higher adiposity and a greater prevalence of cardiometabolic risk factors (Table S1). During a median follow‐up of 5.7 years, 330 incident CVD events were recorded, with a higher incidence in men than in women (12.9% vs. 8.1%, *p* < 0.001). The overall CVD incidence rate was 19.34 per 1000 person‐years, with higher rates in men (23.04) than in women (15.55); sex‐stratified rates for major CVD components were shown in Table S2.

**TABLE 1:** Baseline characteristics of study participants by sex.

Characteristic	Total (*n* = 3168)	Male (*n* = 1548)	Female (*n* = 1620)	*p*
Age, years	72.0 (68.0–76.0)	71.0 (68.0–75.0)	72.00 (68.0–76.0)	0.554
Education level, *n* (%)				< 0.001
No education	694 (21.9)	82 (5.3)	612 (37.8)	
Primary or below	1579 (49.8)	848 (54.8)	731 (45.1)	
Secondary or above	895 (28.3)	618 (39.9)	277 (17.1)	
Smoking status, *n* (%)				< 0.001
Never	2035 (64.2)	567 (36.6)	1468 (90.6)	
Former	899 (28.4)	780 (50.4)	119 (7.3)	
Current	234 (7.4)	201 (13.0)	33 (2.0)	
Alcohol drinking, *n* (%)	437 (13.8)	394 (25.5)	43 (2.7)	< 0.001
Physical activity (PASE)	85.43 (61.19–111.29)	90.64 (60.57–126.56)	84.71 (64.00–102.71)	< 0.001
Energy intake, kcal/day	1748.32 (1404.54–2190.00)	2044.15 (1686.42–2462.90)	1502.15 (1266.25–1841.45)	< 0.001
Diabetes mellitus, *n* (%)	415 (13.1)	202 (13.0)	213 (13.1)	0.976
Hypertension, *n* (%)	1332 (42.0)	670 (43.3)	662 (40.9)	0.180
Cancer history, *n* (%)	142 (4.5)	67 (4.3)	75 (4.6)	0.746
Lipid‐lowering medication, *n* (%)	180 (5.7)	58 (3.7)	122 (7.5)	< 0.001
PBF, %	28.98 (24.19–34.98)	24.52 (21.34–27.38)	34.81 (31.35–37.83)	< 0.001
FMI, kg/m^2^	6.78 (5.34–8.48)	5.59 (4.59–6.65)	8.18 (6.84–9.63)	< 0.001
ALM/weight, %	28.25 (25.38–31.03)	30.91 (29.43–32.53)	25.47 (24.00–27.19)	< 0.001
ALM/height^2^, kg/m^2^	6.56 (5.88–7.24)	7.17 (6.64–7.68)	6.00 (5.53–6.51)	< 0.001
BMI, kg/m^2^	23.36 (21.39–25.57)	23.23 (21.30–25.24)	23.52 (21.52–25.85)	< 0.001
WC, cm	86.36 (80.01–91.44)	86.36 (81.28–92.71)	85.09 (78.74–91.44)	< 0.001
Grip strength, kg	26.00 (22.00–34.00)	34.00 (30.00–38.00)	22.00 (20.00–26.00)	< 0.001
Gait speed, m/s	1.02 (0.88–1.17)	1.07 (0.93–1.22)	0.96 (0.83–1.10)	< 0.001
Five‐times chair stand, s	12.19 (10.16–14.60)	11.95 (10.06–14.25)	12.37 (10.35–14.94)	< 0.001
STS muscle power				
Absolute power, W	143.62 (109.02–183.79)	170.51 (135.87–211.91)	118.97 (93.96–152.24)	< 0.001
Allometric power, W/m^2^	58.05 (46.32–72.47)	64.26 (52.18–77.88)	52.24 (42.13–65.54)	< 0.001
Relative power, W/kg	2.50 (2.04–3.04)	2.77 (2.29–3.33)	2.24 (1.84–2.73)	< 0.001

*Note:* Data were presented as median (interquartile range, IQR) for continuous variables and *n* (%) for categorical variables. Differences between sexes were assessed using the Mann–Whitney *U* test for continuous variables and the *χ* ^2^ test for categorical variables.

Abbreviations: ALM, appendicular lean mass; BMI, body mass index; CVD, cardiovascular disease; FMI, fat mass index; PASE, physical activity scale for the elderly; PBF, percentage of body fat; STS, sit‐to‐stand; WC, waist circumference.

Compared with women, men had higher educational attainment, a higher prevalence of smoking and alcohol drinking, greater physical activity and energy intake (all *p* < 0.001). Regarding body composition, women demonstrated higher adiposity (body fat percentage, FMI and BMI) but lower muscle mass indices than men (all *p* < 0.001). Men demonstrated greater grip strength, faster gait speed and higher muscle power indices (absolute, allometric and relative power) than women (all *p* < 0.001).

### 3.2 Associations of Adiposity, Muscle Strength and Muscle Power With Incident CVD

In fully adjusted models (Model 2, Table 2), higher adiposity measures, including PBF (HR per 1‐SD increase: 1.24, 95% CI: 1.07–1.45), FMI (HR: 1.25, 95% CI: 1.08–1.44), BMI (HR: 1.22, 95% CI: 1.05–1.40) and WC (HR: 1.25, 95% CI: 1.08–1.45), were significantly associated with increased CVD risk in men (all *p* < 0.01). By contrast, no significant associations were observed between any body composition measure and CVD risk in women. After FDR correction, the sex interactions for PBF (*p*_interaction_ = 0.010) and FMI (*p*_interaction_ = 0.020) did not remain significant (both *q* > 0.05).

**TABLE 2:** Sex‐stratified associations of adiposity, muscle strength and muscle power with incident cardiovascular disease (Cox proportional‐hazards models).^a^

Characteristic	Total (*n* = 3168)		Male (*n* = 1548)		Female (*n* = 1620)		*p* for interaction	*q* for interaction
	HR (95% CI)	*p*	HR (95% CI)	*p*	HR (95% CI)	*p*		
No. of incident CVD	330		199		131			
*Model 1*								
Body composition								
PBF, %	1.18 (1.01–1.37)	0.042	1.29 (1.11–1.49)	< 0.001	0.94 (0.80–1.11)	0.461	0.004	0.044
FMI, kg/m^2^	1.20 (1.05–1.37)	0.007	1.31 (1.14–1.50)	< 0.001	1.01 (0.85–1.20)	0.906	0.007	0.044
ALM/weight, %	0.79 (0.68–0.93)	0.006	0.76 (0.66–0.88)	< 0.001	1.00 (0.85–1.18)	0.983	0.014	0.054
ALM/height^2^, kg/m^2^	1.13 (0.99–1.29)	0.071	1.13 (0.98–1.30)	0.093	1.07 (0.90–1.27)	0.426	0.800	0.800
BMI, kg/m^2^	1.19 (1.07–1.33)	0.002	1.28 (1.12–1.48)	< 0.001	1.07 (0.90–1.27)	0.445	0.079	0.153
WC, cm	1.22 (1.09–1.35)	< 0.001	1.31 (1.14–1.51)	< 0.001	1.10 (0.93–1.30)	0.252	0.089	0.153
Muscle strength and function								
Grip strength, kg	0.79 (0.68–0.93)	0.004	0.84 (0.72–0.98)	0.024	0.84 (0.70–1.01)	0.067	0.583	0.776
Gait speed, m/s	0.86 (0.76–0.97)	0.011	0.88 (0.76–1.02)	0.097	0.84 (0.70–1.00)	0.047	0.711	0.776
Five‐times chair stand, s	1.11 (1.01–1.22)	0.035	1.06 (0.93–1.22)	0.383	1.14 (1.01–1.30)	0.042	0.673	0.776
Muscle power								
Absolute power, W	0.94 (0.83–1.07)	0.360	1.02 (0.88–1.18)	0.840	0.80 (0.65–0.98)	0.032	0.058	0.153
Allometric power, W/m^2^	0.95 (0.84–1.07)	0.403	1.03 (0.89–1.19)	0.711	0.82 (0.67–1.00)	0.046	0.073	0.153
Relative power, W/kg	0.86 (0.76–0.97)	0.017	0.92 (0.79–1.07)	0.277	0.77 (0.64–0.94)	0.008	0.127	0.191
*Model 2*								
Body composition								
PBF, %	1.10 (0.94–1.29)	0.251	1.24 (1.07–1.45)	0.006	0.89 (0.75–1.06)	0.191	0.010	0.117
FMI, kg/m^2^	1.12 (0.97–1.28)	0.111	1.25 (1.08–1.44)	0.002	0.94 (0.79–1.13)	0.534	0.020	0.117
ALM/weight, %	0.86 (0.73–1.02)	0.090	0.80 (0.69–0.93)	0.004	1.06 (0.89–1.27)	0.483	0.032	0.128
ALM/height^2^, kg/m^2^	1.07 (0.93–1.23)	0.331	1.09 (0.94–1.26)	0.259	1.01 (0.85–1.21)	0.886	0.698	0.698
BMI, kg/m^2^	1.11 (0.99–1.25)	0.065	1.22 (1.05–1.40)	0.008	0.99 (0.82–1.18)	0.870	0.112	0.187
WC, cm	1.14 (1.02–1.28)	0.023	1.25 (1.08–1.45)	0.003	1.02 (0.85–1.22)	0.844	0.125	0.187
Muscle strength and function								
Grip strength, kg	0.79 (0.67–0.92)	0.003	0.84 (0.72–0.98)	0.026	0.83 (0.69–1.00)	0.049	0.462	0.615
Gait speed, m/s	0.87 (0.77–0.99)	0.032	0.90 (0.77–1.05)	0.166	0.85 (0.71–1.02)	0.088	0.622	0.678
Five‐times chair stand, s	1.10 (1.00–1.22)	0.049	1.06 (0.92–1.21)	0.429	1.15 (1.00–1.32)	0.055	0.551	0.661
Muscle power^b^								
Absolute power, W	0.92 (0.80–1.04)	0.192	0.99 (0.85–1.15)	0.906	0.77 (0.62–0.95)	0.013	0.043	0.129
Allometric power, W/m^2^	0.92 (0.82–1.05)	0.210	1.00 (0.86–1.16)	0.988	0.79 (0.64–0.96)	0.021	0.064	0.153
Relative power, W/kg	0.86 (0.76–0.98)	0.025	0.92 (0.79–1.07)	0.297	0.77 (0.63–0.94)	0.011	0.115	0.187

^Abbreviations: ALM, appendicular lean mass; BMI, body mass index; CI, confidence interval; CVD, cardiovascular disease; FMI, fat mass index; HR, hazard ratio; PBF, percentage of body fat; WC, waist circumference.^

^a^ Values were cause‐specific hazard ratios (95% CI) per 1‐SD increase, estimated from Cox proportional‐hazards models. Model 1 was adjusted for age in the sex‐stratified analyses and additionally adjusted for sex in the total population. Model 2 was additionally adjusted for education level, smoking, alcohol drinking, physical activity, energy intake, diabetes, hypertension, cancer history and lipid‐lowering medication. *p* Values for interaction were derived from models containing the sex × exposure terms. The *q* values were obtained by applying the Benjamini‐Hochberg correction to the sex‐interaction *p* values from Model 1 and Model 2 separately to control the false discovery rate.

^b^ Analyses of five times chair stand time and muscle power were based on 3135 participants (325 events: 196 in 1542 men and 129 in 1593 women), with 33 participants excluded owing to missing data.

Higher grip strength was associated with reduced CVD risk in both men (HR: 0.84) and women (HR: 0.83), with no significant sex interaction (*p* = 0.462). Gait speed and chair stand time showed no associations after full adjustment in either sex. Notably, all muscle power indices, absolute power (HR: 0.77, 95% CI: 0.62–0.95), allometric power (HR: 0.79, 95% CI: 0.64–0.96) and relative power (HR: 0.77, 95% CI: 0.63–0.94), were significantly associated with lower CVD risk in women, but not in men. However, none of the sex interactions remained significant after FDR correction (all *q* interaction > 0.05). RCS regression showed no nonlinear associations in either sex (all *p* for nonlinearity > 0.05; Figure S2). Fine–Gray models were additionally fitted to account for the competing risk of non‐CVD death (Table S6), with results largely consistent with the primary Cox models.

### 3.3 Associations of Regional Fat Distribution With Incident CVD

In fully adjusted models (Model 2, Table 3), trunk adiposity measures showed the strongest associations with CVD risk in men, including trunk FMR (HR per 1‐SD increase: 1.25, 95% CI: 1.07–1.45), trunk FMI (HR: 1.29, 95% CI: 1.11–1.49) and trunk‐to‐total fat ratio (HR: 1.23, 95% CI: 1.05–1.45). Whole‐body FMR was also significantly associated with CVD risk (HR: 1.22). Arm fat measures showed weaker but significant associations (arm FMR: HR 1.19; arm FMI: HR 1.17). In contrast, leg FMR was not consistently associated with CVD risk in men. No significant associations were observed for any regional fat measures in women. After FDR correction, sex interactions for trunk FMR, trunk FMI and whole‐body FMR remained significant (*p*_interaction_ = 0.009, 0.012 and 0.013, respectively; all *q* < 0.05), but not for trunk‐to‐total fat ratio (*p*_interaction_ = 0.034, *q* = 0.086) in Model 2. The proportional‐hazards assumption was met for all main models (Table S7).

**TABLE 3:** Sex‐stratified associations of regional fat distribution measures with incident cardiovascular disease.

Characteristic	Total (*n* = 3168)		Male (*n* = 1548)		Female (*n* = 1620)		*p* for interact ion	*q* for interaction
	HR (95% CI)	*p*	HR (95% CI)	*p*	HR (95% CI)	*p*		
No. of incident CVD	330		199		131			
*Model 1*								
Fat‐to‐muscle ratio (FMR)								
Whole‐body FMR	1.15 (0.98–1.34)	0.080	1.27 (1.10–1.46)	< 0.001	0.96 (0.81–1.13)	0.593	0.004	0.018
Trunk FMR	1.16 (1.02–1.33)	0.028	1.30 (1.13–1.50)	< 0.001	0.96 (0.81–1.14)	0.662	0.003	0.018
Arm FMR	1.17 (0.99–1.39)	0.061	1.23 (1.08–1.40)	0.002	1.03 (0.87–1.22)	0.708	0.014	0.034
Leg FMR	1.02 (0.86–1.20)	0.850	1.13 (0.99–1.29)	0.078	0.93 (0.78–1.10)	0.373	0.051	0.064
Regional fat mass index (FMI)								
Trunk FMI	1.22 (1.08–1.37)	0.001	1.35 (1.17–1.55)	< 0.001	1.02 (0.86–1.21)	0.822	0.006	0.021
Arm FMI	1.17 (1.02–1.35)	0.029	1.23 (1.08–1.40)	0.002	1.04 (0.88–1.23)	0.638	0.031	0.052
Leg FMI	1.08 (0.94–1.25)	0.272	1.17 (1.03–1.34)	0.019	0.97 (0.82–1.15)	0.724	0.041	0.059
Regional‐to‐total fat ratio								
Trunk‐to‐total fat ratio	1.19 (1.06–1.34)	0.004	1.31 (1.13–1.53)	< 0.001	1.02 (0.86–1.20)	0.835	0.031	0.052
Arm‐to‐total fat ratio	1.03 (0.90–1.17)	0.682	0.97 (0.84–1.12)	0.674	1.07 (0.91–1.27)	0.399	0.369	0.369
Leg‐to‐total fat ratio	0.86 (0.76–0.96)	0.010	0.81 (0.70–0.94)	0.005	0.93 (0.79–1.10)	0.406	0.151	0.168
*Model 2*								
Fat‐to‐muscle ratio (FMR)								
Whole‐body FMR	1.08 (0.92–1.27)	0.351	1.22 (1.06–1.42)	0.006	0.91 (0.76–1.09)	0.301	0.013	0.044
Trunk FMR	1.08 (0.94–1.24)	0.272	1.25 (1.07–1.45)	0.004	0.90 (0.75–1.07)	0.232	0.009	0.044
Arm FMR	1.10 (0.92–1.31)	0.294	1.19 (1.03–1.36)	0.014	0.98 (0.82–1.16)	0.781	0.053	0.107
Leg FMR	1.02 (0.86–1.21)	0.812	1.12 (0.97–1.28)	0.118	0.94 (0.79–1.12)	0.495	0.115	0.143
Regional fat mass index (FMI)								
Whole‐body FMI	1.12 (0.97–1.28)	0.111	1.25 (1.08–1.44)	0.002	0.94 (0.79–1.13)	0.534	0.020	0.117
Trunk FMI	1.13 (0.99–1.28)	0.067	1.29 (1.11–1.49)	< 0.001	0.93 (0.78–1.11)	0.427	0.012	0.044
Arm FMI	1.08 (0.93–1.25)	0.296	1.17 (1.02–1.35)	0.021	0.96 (0.80–1.15)	0.649	0.076	0.127
Leg FMI	1.07 (0.92–1.24)	0.370	1.15 (1.00–1.32)	0.044	0.97 (0.81–1.16)	0.729	0.091	0.130
Regional‐to‐total fat ratio								
Trunk‐to‐total fat ratio	1.08 (0.96–1.23)	0.192	1.23 (1.05–1.45)	0.009	0.92 (0.77–1.09)	0.320	0.034	0.086
Arm‐to‐total fat ratio	0.98 (0.85–1.12)	0.727	0.96 (0.83–1.10)	0.553	0.98 (0.83–1.17)	0.846	0.549	0.549
Leg‐to‐total fat ratio	0.94 (0.83–1.07)	0.349	0.87 (0.75–1.01)	0.070	1.05 (0.88–1.25)	0.623	0.188	0.209

*Note:* Values were hazard ratios (95% CI) per 1‐SD increase from Cox proportional‐hazards models. Model 1 was adjusted for age in the sex‐stratified analyses and additionally adjusted for sex in the total population. Model 2 was additionally adjusted for education level, smoking, alcohol drinking, physical activity, energy intake, diabetes, hypertension, cancer history and lipid‐lowering medication. *p* Values for interaction were derived from models containing sex × exposure terms. The *q* values were obtained by applying the Benjamini‐Hochberg correction to the sex‐interaction *p* values from Model 1 and Model 2 separately to control the false discovery rate. FMR = fat mass/lean mass; regional fat mass index = regional fat mass/height^2^; regional‐to‐total fat ratio = regional fat mass/whole‐body fat mass.

Abbreviations: CI, confidence interval; CVD, cardiovascular disease; FMI, fat mass index; FMR, fat‐to‐muscle mass ratio; HR, hazard ratio; SD, standard deviation.

### 3.4 Sex Differences in Regional Fat Distribution

Figure 1 and Table S8 showed that women had significantly higher fat mass index measures in all regions (whole‐body, trunk, arm and leg) and higher FMR measures across all regions (whole‐body, trunk, arm and leg) than men (all *p* < 0.001). Notably, men demonstrated a more centralised fat distribution pattern, characterised by a higher trunk‐to‐total fat ratio (median: 0.564 vs. 0.518, *p* < 0.001), whereas women had higher arm‐to‐total and leg‐to‐total fat ratios (both *p* < 0.001).

**FIGURE 1:**
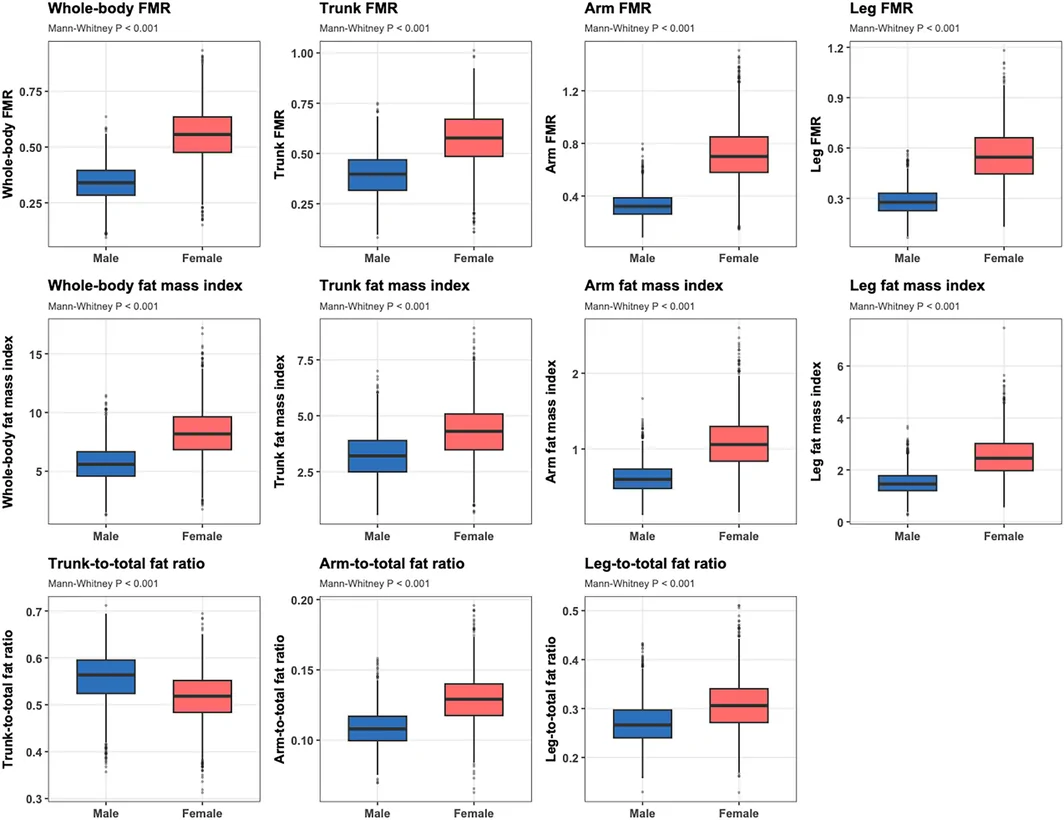
Sex differences in regional fat distribution. Boxplots showed 11 DXA‐derived indicators: Whole‐body, trunk, arm and leg FMR and FMI, as well as trunk‐, arm‐ and leg‐to‐total fat ratios. Boxes indicated median (central line) and interquartile range (box limits). *p* Values were from Mann–Whitney *U* tests comparing men versus women. DXA, dual‐energy x‐ray absorptiometry; FMI, fat mass index; FMR, fat‐to‐muscle ratio.

### 3.5 Correlations of Regional Fat Distribution With Cardiovascular Risk Factors

Partial correlations (Figure 2) showed that in men, measures of central adiposity, including whole‐body FMR, trunk FMR, whole‐body FMI, trunk FMI and trunk‐to‐total fat ratio, were positively correlated with CRP, homocysteine and hypertension (*r* = 0.13–0.22, all *p* < 0.001). Similar patterns were observed for central adiposity in women (all *p* < 0.01). Leg‐to‐total fat ratio was inversely correlated with all four risk factors in both sexes (*r* = −0.09 to −0.21 in men, −0.09 to −0.11 in women; all *p* < 0.05). Additionally, leg FMR and leg FMI were inversely associated with diabetes in women (*r* = −0.10 and −0.09, respectively; both *p* < 0.01).

**FIGURE 2:**
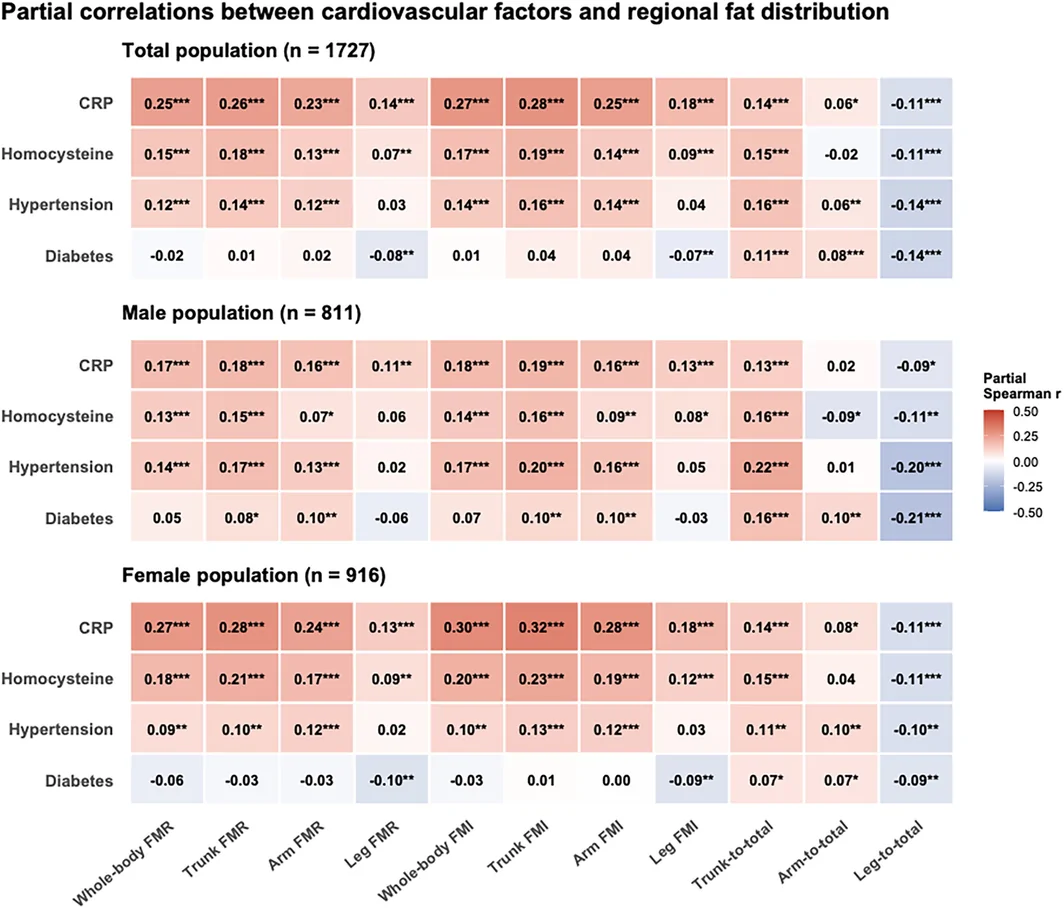
Sex‐stratified partial Spearman's correlation heatmap between cardiovascular risk factors and regional fat distribution measures. Rows showed four cardiovascular risk factors (C‐reactive protein, homocysteine, hypertension and diabetes); columns showed DXA‐derived regional fat measures, comprising the FMR and FMI of the whole body, trunk, arm and leg, as well as the trunk‐to‐total, arm‐to‐total and leg‐to‐total fat ratios. Results were shown for total population (*n* = 1727), men (*n* = 811) and women (*n* = 916). Correlation coefficients were adjusted for age, sex, education, smoking, alcohol drinking, physical activity and energy intake (*n* = 1727 after excluding those with missing CRP or total homocysteine data). Cell colour indicated the direction and magnitude of the partial Spearman correlation coefficient (red: positive; blue: negative). *p < 0.05, **p < 0.01, ***p < 0.001. DXA, dual‐energy x‐ray absorptiometry; FMI, fat mass index; FMR, fat‐to‐muscle ratio.

### 3.6 Sensitivity Analyses

Fine–Gray competing‐risk models (Table S6) and analyses further adding CVD deaths (without prior hospitalisation) to the outcome definition (Tables S9 and S10) yielded results largely unchanged from the primary findings, as did the complete‐case analysis restricted to participants with no missing data (*n* = 3130; Table S11). Further, analyses of major CVD components showed that the associations for CHD and heart failure were largely consistent with the composite CVD outcome (Tables S12–S14). In men, trunk fat measures were positively associated with CHD and heart failure (all *p* < 0.05). In women, higher muscle power was inversely associated with both outcomes (all *p* < 0.05). Associations with stroke were less consistent and did not reach statistical significance. Additional adjustment for blood pressure, creatinine and homocysteine yielded results consistent with the primary findings (Tables S15a–d).

In mutually adjusted models (Table S16), the inverse association of ALM/weight with CVD risk in men was eliminated after adjustment for FMI, whereas whole‐body FMR, trunk FMR and trunk‐to‐total fat ratio remained significantly associated with CVD risk after adjustment for ALM/height^2^ (all *p* < 0.05). Similarly, when STS power was mutually adjusted for FMI and ALM/height^2^, the associations in women remained robust (absolute power: HR 0.75, *p* = 0.012; allometric power: HR 0.77, *p* = 0.019; relative power: HR 0.77, *p* = 0.008) (Table S17). A lag analysis excluding CVD events within the first 2 years yielded consistent findings (Table S18).

## 4 Discussion

In this prospective cohort study of 3168 community‐dwelling older Chinese adults, we observed sex‐stratified associations of adiposity, regional fat distribution and muscle power with incident CVD. In men, higher adiposity was associated with increased CVD risk, whereas in women, higher STS‐derived muscle power was associated with lower CVD risk. However, formal interaction tests did not all remain significant after FDR correction; only trunk FMR, trunk FMI and whole‐body FMR retained significance (*q* interaction < 0.05). These findings suggest that the evidence for effect modification by sex is partial and should be interpreted cautiously. The observed sex‐stratified patterns are exploratory and require validation in independent older cohorts before clinical translation.

The strong and consistent associations between central adiposity and CVD risk in men are consistent with evidence that abdominal adiposity, particularly its visceral component, is a key driver of cardiometabolic disease [30, 31]. Excess visceral adipose tissue (VAT) is characterised by adipose tissue dysfunction, which promotes IR, atherogenic dyslipidaemia and systemic inflammation [32]. Importantly, trunk‐specific measures were more strongly associated with CVD risk than whole‐body adiposity in men, suggesting that fat location matters more than total FM. This is clinically relevant because men preferentially accumulate abdominal fat (android pattern), whereas women deposit fat predominantly in the gluteofemoral region (gynoid pattern) [21, 33].

Despite higher FMI and FMR in women, these regional fat measures were not associated with CVD risk, consistent with the concept that gluteofemoral adipose tissue provides a safe and metabolically favourable depot for lipid storage [19, 21]. Gluteofemoral fat is characterised by lower lipolytic activity and greater capacity for fatty acid uptake, serving as a ‘metabolic sink’ that buffers against the deleterious effects of excess energy [19, 34]. Gluteofemoral adipose is also associated with a beneficial adipokine profile, including higher adiponectin and lower inflammatory cytokines [19]. In contrast, trunk fat, especially VAT, has been prospectively associated with incident CVD independent of generalised adiposity [35]. The positive correlations between trunk FMR, trunk FMI and inflammatory and metabolic risk factors, coupled with the inverse correlations of leg‐to‐total fat ratio with these risk factors, are consistent with the ‘gluteofemoral paradox’ [19]. However, these cross‐sectional correlations preclude causal inference.

It is important to note, however, that DXA‐derived trunk fat includes both visceral and subcutaneous adipose tissue and does not directly quantify intra‐abdominal fat. While recent imaging literature has established the importance of fat distribution for cardiovascular health [36], DXA trunk fat is not interchangeable with directly measured VAT. Therefore, our findings on trunk fat should be interpreted as a composite measure of both visceral and subcutaneous abdominal adiposity, rather than as an isolated indicator of visceral fat alone. Mechanistic inferences drawn from these associations should be considered suggestive and warrant further validation using imaging‐based measures of visceral fat.

In component‐specific analyses, associations for CHD and heart failure were largely consistent with the composite CVD outcome. In men, trunk fat measures were positively associated with both outcomes; in women, muscle power was inversely associated with both outcomes. Associations with stroke were generally non‐significant for most exposures, likely due to limited statistical power from the smaller number of stroke events. Importantly, in mutually adjusted models, whole‐body FMR, trunk FMR and trunk‐to‐total fat ratio remained significantly associated with CVD risk after adjustment for ALM/height^2^, confirming that these associations represent independent fat‐related signals independent of mathematical coupling. By contrast, the protective association of ALM/weight was attenuated after adjustment for FMI, suggesting that it may reflect inverse adiposity rather than a muscle‐specific effect.

Grip strength showed consistent protective associations in both sexes, reinforcing its clinical utility [37] and aligning with the PURE study [15]. In contrast, STS‐derived muscle power was significantly associated with lower CVD risk in women only. Men in our cohort had higher baseline muscle power than women (absolute power: 170.5 vs. 119.0 W, allometric power: 64.3 vs. 52.2 W/m^2^), consistent with well‐established sex differences in muscle function [38]. This may contribute to a ceiling effect, whereby the protective benefit is more detectable in women, who have lower baseline levels and thus greater potential for risk stratification. Additionally, lower‐limb muscle power was particularly relevant to women's mortality [39] and may serve as a more sensitive marker. However, STS performance reflects a broader construct of physical function, influenced by balance, pain, osteoarthritis, cognition, psychological status and frailty, rather than muscle power alone [40]. Given that STS power is also estimated from body mass and height and may partly reflect body size and mobility, we performed sensitivity analyses with mutual adjustment for body composition and a 2‐year lag analysis, which confirmed the robustness of the associations. Nonetheless, the non‐significant sex interactions for muscle power after FDR correction warrant cautious interpretation and future validation.

Our findings extend previous research. Unlike prior studies relying on BMI or BIA, we used DXA for more precise quantification of regional FM and LM [8], enabling distinction between trunk and peripheral fat and revealing their distinct roles in CVD risk. Additionally, while previous studies examined muscle power using laboratory‐based equipment [18], we used the STS muscle power test, a simple alternative that is readily applicable [16, 25]. Moreover, our study simultaneously evaluated sex‐stratified associations across multiple domains within a single Asian cohort, using hard hospitalised CVD endpoints. Finally, while cardiorespiratory fitness has been shown to mitigate obesity‐related risks [25, 41], our exploratory findings suggest a potential association between muscle power and lower CVD risk in women. However, we did not test an adiposity‐by‐power interaction and therefore cannot determine whether muscle power counteracts obesity‐related risk in women, a question requiring further investigation.

Our findings have several clinical implications. First, the sex‐stratified patterns underscore the potential value of sex‐stratified cardiometabolic risk assessment in older adults. Trunk fat and STS‐derived muscle power may hold potential for improving risk stratification, although prospective validation is needed. Second, the strong association between central adiposity and CVD risk in men generates the hypothesis that interventions targeting abdominal fat might be particularly beneficial; similarly, the protective association of muscle power in women suggests a potential role for resistance or power training in this group, both hypotheses that warrant testing in randomised trials. Third, the STS muscle power test could be easily integrated into routine geriatric assessments to identify older women at increased CVD risk who may benefit from targeted exercise interventions [42]. Fourth, the inverse association of leg‐to‐total fat ratio with metabolic risk factors suggests that maintaining lower‐body fat distribution may be metabolically favourable, particularly, in women [43]. All these implications are exploratory and require confirmation in future interventional studies.

Strengths of this study included DXA‐based body composition, simultaneous evaluation of overall and regional adiposity with muscle strength and power, a sex‐balanced cohort and robust sensitivity analyses. Several limitations should also be acknowledged. First, the observational design precludes causal inferences. Second, although we adjusted for potential confounders, residual confounding by unmeasured or incompletely measured factors (e.g., socioeconomic status) cannot be ruled out. Third, the STS test, while practical, provides an indirect estimate of muscle power; direct measurement using force plates may be more accurate [44]. Fourth, although the CMS database covers over 95% of hospital admissions in Hong Kong, it may still miss CVD events managed entirely in outpatient or primary care settings. Notably, women had higher lipid‐lowering medication use than men, suggesting more outpatient care, which may have attenuated true associations in women and partly explain null findings.

Fifth, the composite CVD outcome comprises conditions of varying severity and event numbers for component‐specific analyses were limited, especially for stroke. Sixth, baseline medical history, including prior diagnoses of CVD, was based on self‐report, which may introduce recall bias. Seventh, DXA‐derived trunk fat cannot distinguish visceral from subcutaneous fat and is not interchangeable with CT/MRI; similarly, LM is a proxy for skeletal muscle mass. Our mechanistic inferences are therefore preliminary and require confirmation by direct visceral fat imaging. Eighth, body composition and muscle power were assessed only at baseline and may have changed over time due to aging and frailty, potentially leading to regression dilution bias. Although a 2‐year lag analysis to mitigate reverse causation yielded consistent estimates, the absence of serial measurements precludes full correction for this bias. Ninth, despite FDR correction, several significant sex interactions were attenuated. Our sex‐stratified findings should thus be considered exploratory. Finally, our cohort consisted of older Chinese adults living in Hong Kong and the generalizability of our findings to other ethnic groups or younger populations requires confirmation.

## 5 Conclusions

In this prospective cohort study of older Chinese adults, we observed sex‐stratified associations of adiposity, regional fat distribution and muscle power with incident CVD. Higher adiposity and lower relative muscle mass were associated with higher CVD risk in men only, whereas higher muscle power was associated with lower risk in women only. Central adiposity, particularly trunk fat, was associated with CVD risk in men, whereas peripheral (leg) fat distribution was associated with a favourable metabolic profile in both sexes. These exploratory findings highlight the importance of considering both fat distribution and sex in CVD risk assessment in older adults. The simple and low‐cost STS muscle power test, together with DXA‐derived regional fat measures, may be useful for potential risk identification and guiding hypothesis‐driven research. Future studies should investigate the metabolic mechanisms underlying these sex‐stratified patterns and evaluate whether interventions targeting central adiposity in men and muscle power in women translate into reduced cardiovascular risk in older populations.

## Author Contributions

**Ting Zhang:** conceptualisation, methodology, formal analysis and visualisation, writing – original draft, writing – review and editing. **Yuan Gao:** methodology, formal analysis and visualisation, writing – review and editing. **Yafei Wu:** methodology, writing – review and editing. **Jason Leung:** data curation, writing – review and editing. **Timothy Kwok:** conceptualisation, resources, writing – review and editing, supervision, project administration. All authors had read and approved the final version of this manuscript.

## Funding

This study was funded by the National Institutes of Health R01 grant (AR049439–01A1) and the Research Grants Council Earmarked grant (CUHK4101/02M).

## Ethics Statement

The study was conducted according to the guidelines of the Declaration of Helsinki and approved by the joint Chinese University of Hong Kong and New Territories East Cluster Clinical Research Ethics Committees in Hong Kong (Reference number [2003.102]).

## Consent

Written informed consent was obtained from all participants.

## Conflicts of Interest

The authors declare no conflicts of interest.

## Supporting information


**Figure S1:** Flowchart of the study participants. ALM, appendicular lean mass; CVD, cardiovascular disease; FMI, fat mass index; STS, sit‐to‐stand.
**Figure S2:** Multivariable‐adjusted restricted cubic spline (RCS) plots for the associations of adiposity, muscle strength and muscle power with CVD incidence. (A) male; (B) female. Knots were placed at the 10th, 50th and 90th percentiles of each exposure variable, with the median value serving as the reference (HR = 1). The solid lines represented the fully adjusted HRs and the shaded areas indicated 95% CIs. All models were adjusted for age, sex, education level, smoking, alcohol drinking, physical activity, energy intake, diabetes, hypertension, cancer history and lipid‐lowering medication. AlloP, allometric power; ALM, appendicular lean mass; AP, absolute power; BMI, body mass index; CVD, cardiovascular disease; FMI, fat mass index; GS, grip strength; ht, height; PBF, percentage of body fat; RP, relative power; STS, sit‐to‐stand; WC, waist circumference; wt, weight.
**Table S1:** Baseline characteristics of participants excluded for prevalent CVD at baseline (*n* = 832) compared with those retained in the main analyses (*n* = 3168).
**Table S2:** Incident cardiovascular events by subtype and sex, excluding participants with baseline disease for each condition.
**Table S3:** Missing data for all exposures, covariates and outcomes in the main analytic cohort (*n* = 3168).
**Table S4:** Sex‐stratified means and standard deviations of exposure variables, corresponding to the per‐1‐SD hazard ratios reported in the main text.
**Table S5:** Statistical software, packages and functions used.
**Table S6:** Sex‐stratified associations of adiposity, muscle strength and muscle power with incident CVD (Fine‐Gray competing‐risk models).
**Table S7:** Assessment of the proportional‐hazards assumption for all exposures.
**Table S8:** Sex differences in regional fat distribution.
**Table S9:** Sensitivity analysis adding CVD deaths (without prior CVD hospitalisation) to the incident CVD outcome definition (Cox model).
**Table S10:** Sensitivity analysis adding CVD deaths (without prior CVD hospitalisation) to the incident CVD outcome definition (Fine–Gray model).
**Table S11:** Complete‐case analysis restricted to participants with complete data on all exposures, covariates and outcomes (*n* = 3130).
**Table S12:** Sex‐stratified associations of body composition, muscle strength, muscle power and regional fat distribution with incident CHD.
**Table S13:** Sex‐stratified associations of body composition, muscle strength, muscle power and regional fat distribution with incident stroke.
**Table S14:** Sex‐stratified associations of body composition, muscle strength, muscle power and regional fat distribution with incident heart failure.
**Table S15a:** Sex‐stratified associations with incident CVD after additional adjustment for measured blood pressure, serum creatinine and homocysteine.
**Table S15b:** Sex‐stratified associations with incident CHD after additional adjustment for measured blood pressure, serum creatinine and homocysteine.
**Table S15c:** Sex‐stratified associations with incident stroke after additional adjustment for measured blood pressure, serum creatinine and homocysteine.
**Table S15d:** Sex‐stratified associations with incident heart failure after additional adjustment for measured blood pressure, serum creatinine and homocysteine.
**Table S16:** Mutually adjusted models for ratio‐based body composition measures, adjusted for standardised adiposity or muscle indices.
**Table S17:** Sex‐stratified mutually adjusted associations of STS‐derived muscle power with CVD risk, additionally adjusted for adiposity (FMI) and muscle mass (ALM/height^2^).
**Table S18:** Sex‐stratified associations of STS‐derived muscle power with CVD risk after excluding cardiovascular events within the first 2 years of follow‐up.

## Data Availability

The data that support the findings of this study are available from the corresponding author upon reasonable request.
