# Supplementary material for: Sex‐Stratified Associations of DXA‐Derived Adiposity, Regional Fat Distribution and Muscle Power With Incident Hospitalised Cardiovascular Disease in Older Adults: A Prospective Cohort Study
Source: Diabetes Obes Metab. 2026 Sep 14;28(12):11418–31. doi: 10.1111/dom.71360 (PMC13730981; doi:10.1111/dom.71360)
Supplement: Supplementary file 1 [file DOM-28-11418-s001.docx]

**Supplementary Appendix**

**List of supplementary materials**

| **Contents** | **Page** |
| --- | --- |
| **Figure S1.** Flowchart of the study participants. | 3 |
| **Figure S2.** Multivariable-adjusted restricted cubic spline (RCS) plots for the associations of adiposity, muscle strength and muscle power with CVD incidence. | 4 |
| **Table S1.** Baseline characteristics of participants excluded for prevalent CVD at baseline (n = 832) compared with those retained in the main analyses (n = 3,168). | 5 |
| **Table S2.** Incident cardiovascular events by subtype and sex, excluding participants with baseline disease for each condition. | 6 |
| **Table S3.** Missing data for all exposures, covariates and outcomes in the main analytic cohort (n = 3,168). | 7-8 |
| **Table S4.** Sex-stratified means and standard deviations of exposure variables, corresponding to the per-1-SD hazard ratios reported in the main text. | 9-10 |
| **Table S5.** Statistical software, packages and functions used. | 11 |
| **Table S6.** Sex-stratified associations of adiposity, muscle strength and muscle power with incident CVD (Fine-Gray competing-risk models). | 12-13 |
| **Table S7.** Assessment of the proportional-hazards assumption for all exposures. | 14-20 |
| **Table S8.** Sex differences in regional fat distribution. | 21 |
| **Table S9.** Sensitivity analysis adding CVD deaths (without prior CVD hospitalization) to the incident CVD outcome definition (Cox model). | 22-23 |
| **Table S10.** Sensitivity analysis adding CVD deaths (without prior CVD hospitalization) to the incident CVD outcome definition (Fine-gray model). | 24-25 |
| **Table S11.** Complete-case analysis restricted to participants with complete data on all exposures, covariates, and outcomes (n = 3,130). | 26-28 |
| **Table S12.** Sex-stratified associations of body composition, muscle strength, muscle power and regional fat distribution with incident CHD. | 29-31 |
| **Table S13.** Sex-stratified associations of body composition, muscle strength, muscle power and regional fat distribution with incident stroke. | 32-34 |
| **Table S14.** Sex-stratified associations of body composition, muscle strength, muscle power and regional fat distribution with incident heart failure. | 35-37 |
| **Table S15a.** Sex-stratified associations with incident CVD after additional adjustment for measured blood pressure, serum creatinine and homocysteine. | 38-39 |
| **Table S15b.** Sex-stratified associations with incident CHD after additional adjustment for measured blood pressure, serum creatinine and homocysteine. | 40-41 |
| **Table S15c.** Sex-stratified associations with incident stroke after additional adjustment for measured blood pressure, serum creatinine and homocysteine. | 42-43 |
| **Table S15d.** Sex-stratified associations with incident heart failure after additional adjustment for measured blood pressure, serum creatinine and homocysteine. | 44-45 |
| **Table S16.** Mutually adjusted models for ratio-based body composition measures, adjusted for standardized adiposity or muscle indices. | 46-47 |
| **Table S17.** Sex-stratified mutually adjusted associations of STS-derived muscle power with CVD risk, additionally adjusted for adiposity (FMI) and muscle mass (ALM/height²). | 48 |
| **Table S18.** Sex-stratified associations of STS-derived muscle power with CVD risk after excluding cardiovascular events within the first two years of follow-up. | 49 |

**Figure S1** Flowchart of the study participants. Abbreviations: CVD, cardiovascular disease; STS, sit-to-stand; FMI, fat mass index; ALM, appendicular lean mass.

**Supplementary Figure S2. Multivariable-adjusted restricted cubic spline (RCS) plots for the associations of adiposity, muscle strength and muscle power with CVD incidence.** (A) male; (B) female. Knots were placed at the 10th, 50th and 90th percentiles of each exposure variable, with the median value serving as the reference (HR=1). The solid lines represented the fully adjusted HRs, and the shaded areas indicated 95% CIs. All models were adjusted for adjusted for age, education level, smoking, alcohol drinking, physical activity, energy intake, diabetes, hypertension, cancer history and lipid-lowering medication. CVD, cardiovascular disease; PBF, percentage of body fat; FMI, fat mass index; ALM, appendicular lean mass; wt, weight; ht, height; BMI, body mass index; WC, waist circumference; GS, grip strength; STS, sit-to-stand; AP, absolute power; AlloP, allometric power; RP, relative power.

**Supplementary Table S1. Baseline characteristics of participants excluded for prevalent CVD at baseline (n = 832) compared with those retained in the main analyses (n = 3,168).**

| **Characteristic** | **Presentation** | **Included in main analyses**  **(n = 3,168)** | **Excluded for prevalent CVD (n = 832)** | **P** |
| --- | --- | --- | --- | --- |
| Age, years | median (IQR) | 72.00 (68.00-76.00) | 72.00 (69.00-76.00) | 0.001 |
| BMI, kg/m² | median (IQR) | 23.36 (21.39-25.57) | 24.23 (22.07-26.54) | <0.001 |
| WC, cm | median (IQR) | 86.36 (80.01-91.44) | 88.90 (82.55-95.25) | <0.001 |
| PBF, % | median (IQR) | 28.98 (24.19-34.98) | 29.77 (25.28-35.59) | 0.011 |
| FMI, kg/m² | median (IQR) | 6.78 (5.34-8.48) | 7.21 (5.74-8.85) | <0.001 |
| ALM/weight, % | median (IQR) | 28.25 (25.38-31.03) | 27.96 (25.03-30.63) | 0.008 |
| ALM/height², kg/m² | median (IQR) | 6.56 (5.88-7.24) | 6.69 (6.04-7.45) | <0.001 |
| Grip strength, kg | median (IQR) | 26.00 (22.00-34.00) | 28.00 (22.00-34.00) | 0.893 |
| Gait speed, m/s | median (IQR) | 1.02 (0.88-1.17) | 0.97 (0.84-1.11) | <0.001 |
| Five-times chair stand, s | median (IQR) | 12.19 (10.16-14.60) | 12.69 (10.58-15.44) | <0.001 |
| Absolute power, W | median (IQR) | 143.62 (109.02-183.79) | 143.52 (109.69-188.30) | 0.652 |
| Physical activity (PASE) | median (IQR) | 85.43 (61.19-111.29) | 84.71 (59.71-110.71) | 0.056 |
| Energy intake, kcal/day | median (IQR) | 1748.32 (1404.54-2190.00) | 1766.84 (1439.18-2193.01) | 0.535 |
| Women | n (%) | 1620 (51.1) | 380 (45.7) | 0.006 |
| Secondary education or above | n (%) | 2474 (78.1) | 670 (80.5) | 0.140 |
| Current smoker | n (%) | 899 (28.4) | 292 (35.1) | <0.001 |
| Alcohol drinking | n (%) | 437 (13.8) | 85 (10.2) | 0.008 |
| Diabetes | n (%) | 415 (13.1) | 175 (21.0) | <0.001 |
| Hypertension (self-reported) | n (%) | 1332 (42.0) | 620 (74.5) | <0.001 |
| Cancer history | n (%) | 142 (4.5) | 35 (4.2) | 0.803 |
| Lipid-lowering medication | n (%) | 180 (5.7) | 180 (21.6) | <0.001 |

Continuous variables are presented as medians (IQR) and were compared using the Mann-Whitney U test; categorical variables as numbers (%) and were compared using the chi-squared test. Abbreviations: IQR, interquartile range; ALM, appendicular lean mass; BMI, body mass index; CVD, cardiovascular disease; FMI, fat mass index; FMR, fat-to-muscle ratio; PASE, Physical Activity Scale for the Elderly; PBF, percentage of body fat; WC, waist circumference.

**Supplementary Table S2.** Incident cardiovascular events by subtype and sex, excluding participants with baseline disease for each condition.

| **Outcome** | **Sex** | **No. at risk** | **Events** | **Competing deaths** | **Person-years** | **Median follow-up, y** | **Incidence rate per 1000 person-years (95% CI)** |
| --- | --- | --- | --- | --- | --- | --- | --- |
| CVD | Total | 3,168 | 330 | 142 | 17,063 | 5.67 | 19.34 (17.31, 21.54) |
|  | Men | 1,548 | 199 | 97 | 8,638 | 6.00 | 23.04 (19.95, 26.47) |
|  | Women | 1,620 | 131 | 45 | 8,425 | 5.22 | 15.55 (13.00, 18.45) |
| CHD | Total | 3,408 | 177 | 183 | 18,796 | 5.72 | 9.42 (8.08, 10.91) |
|  | Men | 1,683 | 113 | 126 | 9,647 | 6.04 | 11.71 (9.65, 14.08) |
|  | Women | 1,725 | 64 | 57 | 9,148 | 5.24 | 7.00 (5.39, 8.93) |
| Stroke | Total | 3,825 | 150 | 214 | 21,257 | 5.73 | 7.06 (5.97, 8.28) |
|  | Men | 1,891 | 94 | 151 | 10,934 | 6.04 | 8.60 (6.95, 10.52) |
|  | Women | 1,934 | 56 | 63 | 10,322 | 5.24 | 5.43 (4.10, 7.04) |
| Heart failure | Total | 3,849 | 130 | 218 | 21,414 | 5.74 | 6.07 (5.07, 7.21) |
|  | Men | 1,927 | 77 | 157 | 11,169 | 6.05 | 6.89 (5.44, 8.62) |
|  | Women | 1,922 | 53 | 61 | 10,245 | 5.24 | 5.17 (3.88, 6.77) |

For each outcome, participants with the corresponding baseline condition were excluded (CVD: n = 832; CHD: n = 592; stroke: n = 175; heart failure: n = 151). Because exclusions are condition-specific, the at-risk population differs across outcomes. Abbreviations: CI, confidence interval; CVD, cardiovascular disease; CHD, coronary heart disease.

**Supplementary Table S3.** Missing data for all exposures, covariates and outcomes in the main analytic cohort (n = 3,168).

| **Variable** | **No. missing (%)** |
| --- | --- |
| ***Exposures*** |  |
| PBF, % | 0 (0.00%) |
| FMI, kg/m² | 0 (0.00%) |
| ALM/weight, % | 0 (0.00%) |
| ALM/height², kg/m² | 0 (0.00%) |
| BMI, kg/m² | 0 (0.00%) |
| WC, cm | 1 (0.03%) |
| Grip strength, kg | 0 (0.00%) |
| Gait speed, m/s | 0 (0.00%) |
| Five-times chair stand, s | 33 (1.04%) |
| Absolute power, W | 33 (1.04%) |
| Allometric power, W/m² | 33 (1.04%) |
| Relative power, W/kg | 33 (1.04%) |
| Whole-body FMR | 0 (0.00%) |
| Trunk FMR | 0 (0.00%) |
| Arm FMR | 0 (0.00%) |
| Leg FMR | 0 (0.00%) |
| Whole-body FMI | 0 (0.00%) |
| Trunk FMI | 0 (0.00%) |
| Arm FMI | 0 (0.00%) |
| Leg FMI | 0 (0.00%) |
| Trunk-to-total fat ratio | 0 (0.00%) |
| Arm-to-total fat ratio | 0 (0.00%) |
| Leg-to-total fat ratio | 0 (0.00%) |
| ***Covariates and outcome*** |  |
| Age, years | 0 (0.00%) |
| Sex | 0 (0.00%) |
| Education level | 0 (0.00%) |
| Smoking status | 0 (0.00%) |
| Alcohol drinking | 1 (0.03%) |
| Physical activity (PASE) | 0 (0.00%) |
| Energy intake, kcal/day | 3 (0.09%) |
| Diabetes | 0 (0.00%) |
| Hypertension (self-reported) | 0 (0.00%) |
| Cancer history | 0 (0.00%) |
| Lipid-lowering medication | 0 (0.00%) |
| Incident CVD (outcome) | 0 (0.00%) |
| Follow-up time | 0 (0.00%) |

Of the 36 variables used, 29 were complete for all participants. Missing data were confined to waist circumference (n = 1), the five-times chair stand test (n = 33; muscle power indices derived from it shared the same missingness), energy intake (n = 3), and alcohol drinking (n = 1). Chair-stand and waist circumference were not imputed; analyses involving these exposures were performed on complete cases. Energy intake and alcohol drinking were completed with the sex-specific median and the modal value, respectively, affecting 4 of 3,168 participants (0.13%). A complete-case analysis restricted to participants with no missing values on any variable (n = 3,130) yielded materially unchanged estimates (Supplementary Table S11).

Abbreviations: ALM, appendicular lean mass; BMI, body mass index; CVD, cardiovascular disease; FMI, fat mass index; FMR, fat-to-muscle ratio; PASE, Physical Activity Scale for the Elderly; PBF, percentage of body fat; WC, waist circumference.

**Supplementary Table S4.** Sex-stratified means and standard deviations of exposure variables, corresponding to the per-1-SD hazard ratios reported in the main text.

| **Exposure variable** | **Overall (n = 3,168)** | **Men (n = 1,548)** | **Women (n = 1,620)** |
| --- | --- | --- | --- |
| ***Body composition*** |  |  |  |
| PBF, % | 29.3 ± 7.2 | 24.0 ± 4.9 | 34.3 ± 5.3 |
| FMI, kg/m² | 6.97 ± 2.38 | 5.62 ± 1.68 | 8.27 ± 2.23 |
| ALM/weight, % | 28.3 ± 3.7 | 31.1 ± 2.4 | 25.6 ± 2.5 |
| ALM/height², kg/m² | 6.60 ± 0.95 | 7.18 ± 0.81 | 6.04 ± 0.72 |
| BMI, kg/m² | 23.5 ± 3.3 | 23.2 ± 3.1 | 23.7 ± 3.4 |
| WC, cm | 85.9 ± 9.2 | 86.6 ± 8.9 | 85.2 ± 9.5 |
| ***Muscle strength and function*** |  |  |  |
| Grip strength, kg | 28.1 ± 8.1 | 34.1 ± 6.8 | 22.4 ± 4.4 |
| Gait speed, m/s | 1.02 ± 0.23 | 1.08 ± 0.23 | 0.96 ± 0.21 |
| Five-times chair stand, s | 12.9 ± 4.4 | 12.5 ± 3.7 | 13.3 ± 5.0 |
| ***Muscle power*** |  |  |  |
| Absolute power, W | 151.6 ± 59.8 | 178.6 ± 60.8 | 125.5 ± 45.5 |
| Allometric power, W/m² | 60.7 ± 20.9 | 66.8 ± 21.2 | 54.8 ± 18.7 |
| Relative power, W/kg | 2.59 ± 0.83 | 2.87 ± 0.84 | 2.31 ± 0.71 |
| ***Fat-to-muscle ratio (FMR)*** |  |  |  |
| Whole-body FMR | 0.449 ± 0.154 | 0.337 ± 0.087 | 0.555 ± 0.125 |
| Trunk FMR | 0.482 ± 0.161 | 0.388 ± 0.117 | 0.573 ± 0.145 |
| Arm FMR | 0.527 ± 0.262 | 0.327 ± 0.103 | 0.718 ± 0.223 |
| Leg FMR | 0.423 ± 0.187 | 0.282 ± 0.081 | 0.557 ± 0.157 |
| ***Regional fat mass index (FMI)*** |  |  |  |
| Whole-body FMI | 6.97 ± 2.38 | 5.62 ± 1.68 | 8.27 ± 2.23 |
| Trunk FMI | 3.75 ± 1.31 | 3.17 ± 1.09 | 4.29 ± 1.28 |
| Arm FMI | 0.853 ± 0.379 | 0.613 ± 0.209 | 1.082 ± 0.363 |
| Leg FMI | 2.03 ± 0.83 | 1.50 ± 0.47 | 2.53 ± 0.79 |
| ***Regional-to-total fat ratio*** |  |  |  |
| Trunk-to-total fat ratio | 0.536 ± 0.059 | 0.556 ± 0.057 | 0.516 ± 0.054 |
| Arm-to-total fat ratio | 0.119 ± 0.019 | 0.108 ± 0.013 | 0.129 ± 0.018 |
| Leg-to-total fat ratio | 0.289 ± 0.053 | 0.270 ± 0.045 | 0.308 ± 0.054 |

Values were means ± SD. Values for chair-stand time and muscle power indices were based on 3,135 participants with complete chair stand data; waist circumference on 3,167 participants. Abbreviations: ALM, appendicular lean mass; BMI, body mass index; FMI, fat mass index; FMR, fat-to-muscle ratio; PBF, percentage of body fat; SD, standard deviation; WC, waist circumference.

**Supplementary Table S5.** Statistical software, packages and functions used.

| **Software or package** | **Version** | **Functions used** |
| --- | --- | --- |
| R | 4.3.3 | Base environment for all analyses |
| survival | 3.5.8 | coxph() for Cox proportional-hazards models; cox.zph() for tests of the proportional-hazards assumption based on scaled Schoenfeld residuals; Surv() for the survival object; anova() on nested coxph objects for likelihood ratio tests of the sex×exposure interaction |
| readxl | 1.4.3 | read_excel() for data import |
| dplyr | 1.1.4 | Data management and derivation of exposure variables |
| cmprsk | 2.2.11 | crr() for Fine-Gray subdistribution hazard models treating deaths from other causes as competing events |
| stats (base R) | 4.3.3 | scale() for standardisation to 1-SD units; p.adjust(method = “BH”) for control of the false discovery rate; poisson.test() for exact confidence intervals of incidence rates; t.test() and chisq.test() for baseline comparisons |

All analyses were performed in R. Cox proportional-hazards models were fitted with coxph() using the Efron method for tied event times. Exposures were standardised with scale() so that hazard ratios were expressed per 1-SD increase. Tests of effect modification by sex were likelihood ratio tests comparing nested models with and without a sex×exposure product term, obtained with anova() applied to two coxph objects. The false discovery rate was controlled with p.adjust(method = “BH”). The proportional-hazards assumption was assessed with cox.zph(), which is based on scaled Schoenfeld residuals, for the exposure and for the model as a whole. Abbreviations: BH, Benjamini–Hochberg; HR, hazard ratio; PH, proportional hazards.

**Supplementary Table S6** Sex-stratified associations of adiposity, muscle strength and muscle power with incident CVD (Fine-Gray competing-risk models).

|  | **Total** | | **Male** | | **Female** | |
| --- | --- | --- | --- | --- | --- | --- |
| **Characteristic** | **HR (95% CI)** | **P** | **HR (95% CI)** | **P** | **HR (95% CI)** | **P** |
| **Model 1** |  |  |  |  |  |  |
| ***Body composition*** |  |  |  |  |  |  |
| PBF, % | 1.180 (1.004-1.387) | 0.044 | 1.297 (1.122-1.500) | <0.001 | 0.942 (0.796-1.116) | 0.490 |
| FMI, kg/m² | 1.198 (1.049-1.367) | 0.008 | 1.315 (1.148-1.507) | <0.001 | 1.007 (0.849-1.195) | 0.930 |
| ALM/weight, % | 0.795 (0.673-0.939) | 0.007 | 0.761 (0.660-0.877) | <0.001 | 1.000 (0.840-1.191) | 1.000 |
| ALM/height², kg/m² | 1.135 (0.987-1.305) | 0.077 | 1.136 (0.976-1.322) | 0.100 | 1.067 (0.902-1.263) | 0.450 |
| BMI, kg/m² | 1.189 (1.066-1.327) | 0.002 | 1.291 (1.119-1.489) | <0.001 | 1.064 (0.901-1.256) | 0.470 |
| WC, cm | 1.210 (1.083-1.352) | <0.001 | 1.307 (1.125-1.519) | <0.001 | 1.095 (0.929-1.291) | 0.280 |
| ***Muscle strength and function*** |  |  |  |  |  |  |
| Grip strength, kg | 0.805 (0.692-0.936) | 0.005 | 0.852 (0.736-0.985) | 0.030 | 0.841 (0.706-1.003) | 0.054 |
| Gait speed, m/s | 0.864 (0.768-0.973) | 0.016 | 0.888 (0.764-1.033) | 0.120 | 0.839 (0.705-1.000) | 0.049 |
| Five-times chair stand, s | 1.107 (1.008-1.216) | 0.033 | 1.062 (0.916-1.231) | 0.430 | 1.143 (1.016-1.286) | 0.026 |
| ***Muscle power*** |  |  |  |  |  |  |
| Absolute power, W | 0.945 (0.834-1.071) | 0.380 | 1.020 (0.888-1.171) | 0.780 | 0.803 (0.647-0.995) | 0.045 |
| Allometric power, W/m² | 0.953 (0.847-1.072) | 0.430 | 1.033 (0.902-1.182) | 0.640 | 0.818 (0.664-1.008) | 0.059 |
| Relative power, W/kg | 0.862 (0.763-0.973) | 0.016 | 0.922 (0.799-1.064) | 0.260 | 0.775 (0.642-0.937) | 0.008 |
| **Model 2** |  |  |  |  |  |  |
| ***Body composition*** |  |  |  |  |  |  |
| PBF, % | 1.103 (0.932-1.306) | 0.250 | 1.252 (1.077-1.456) | 0.004 | 0.890 (0.741-1.068) | 0.210 |
| FMI, kg/m² | 1.119 (0.972-1.287) | 0.120 | 1.260 (1.092-1.453) | 0.002 | 0.938 (0.779-1.130) | 0.500 |
| ALM/weight, % | 0.866 (0.729-1.029) | 0.100 | 0.797 (0.686-0.926) | 0.003 | 1.066 (0.888-1.280) | 0.490 |
| ALM/height², kg/m² | 1.074 (0.932-1.238) | 0.330 | 1.094 (0.940-1.273) | 0.250 | 1.008 (0.844-1.205) | 0.930 |
| BMI, kg/m² | 1.113 (0.991-1.250) | 0.071 | 1.223 (1.054-1.418) | 0.008 | 0.978 (0.814-1.174) | 0.810 |
| WC, cm | 1.134 (1.007-1.278) | 0.038 | 1.249 (1.065-1.464) | 0.006 | 1.006 (0.840-1.205) | 0.950 |
| ***Muscle strength and function*** |  |  |  |  |  |  |
| Grip strength, kg | 0.796 (0.680-0.931) | 0.004 | 0.848 (0.729-0.988) | 0.034 | 0.830 (0.694-0.993) | 0.042 |
| Gait speed, m/s | 0.877 (0.775-0.993) | 0.039 | 0.898 (0.767-1.052) | 0.180 | 0.856 (0.709-1.033) | 0.110 |
| Five-times chair stand, s | 1.105 (1.003-1.217) | 0.044 | 1.058 (0.912-1.228) | 0.450 | 1.142 (1.005-1.298) | 0.041 |
| ***Muscle power*** |  |  |  |  |  |  |
| Absolute power, W | 0.918 (0.807-1.045) | 0.190 | 0.993 (0.860-1.147) | 0.930 | 0.767 (0.612-0.961) | 0.021 |
| Allometric power, W/m² | 0.926 (0.821-1.045) | 0.210 | 1.004 (0.874-1.154) | 0.950 | 0.787 (0.633-0.977) | 0.030 |
| Relative power, W/kg | 0.866 (0.766-0.979) | 0.022 | 0.921 (0.797-1.066) | 0.270 | 0.777 (0.639-0.944) | 0.011 |

Values were subdistribution hazard ratios (95% CI) per 1-SD increase, estimated from Fine-Gray models with non-CVD death as the competing event. Model 1 was adjusted for age in the sex-stratified analyses and additionally adjusted for sex in the total population. Model 2 was additionally adjusted for education level, smoking, alcohol drinking, physical activity, energy intake, diabetes, hypertension, cancer history and lipid-lowering medication use. Abbreviations: HR, hazard ratio; CI, confidence interval; CVD, cardiovascular disease; PBF, percentage of body fat; FMI, fat mass index; ALM, appendicular lean mass; BMI, body mass index; WC, waist circumference.

**Supplementary Table S7.** Assessment of the proportional-hazards assumption for all exposures.

| **Exposure** | **Sex** | **No. at risk** | **Events** | **P for the exposure** | **P for the global test** |
| --- | --- | --- | --- | --- | --- |
| **Any CVD hospitalisation** |  |  |  |  |  |
| PBF, % | Men | 1,548 | 199 | 0.773 | 0.226 |
|  | Women | 1,620 | 131 | 0.722 | 0.580 |
| FMI, kg/m² | Men | 1,548 | 199 | 0.559 | 0.218 |
|  | Women | 1,620 | 131 | 0.815 | 0.601 |
| ALM/weight, % | Men | 1,548 | 199 | 0.256 | 0.155 |
|  | Women | 1,620 | 131 | 0.546 | 0.566 |
| ALM/height², kg/m² | Men | 1,548 | 199 | 0.843 | 0.219 |
|  | Women | 1,620 | 131 | 0.663 | 0.599 |
| BMI, kg/m² | Men | 1,548 | 199 | 0.532 | 0.228 |
|  | Women | 1,620 | 131 | 0.916 | 0.612 |
| WC, cm | Men | 1,548 | 199 | 0.614 | 0.211 |
|  | Women | 1,619 | 131 | 0.261 | 0.484 |
| Grip strength, kg | Men | 1,548 | 199 | 0.757 | 0.198 |
|  | Women | 1,620 | 131 | 0.246 | 0.432 |
| Gait speed, m/s | Men | 1,548 | 199 | 0.756 | 0.204 |
|  | Women | 1,620 | 131 | 0.659 | 0.609 |
| Five-times chair stand, s | Men | 1,542 | 196 | 0.808 | 0.269 |
|  | Women | 1,593 | 129 | 0.126 | 0.404 |
| Absolute power, W | Men | 1,542 | 196 | 0.797 | 0.278 |
|  | Women | 1,593 | 129 | 0.405 | 0.541 |
| Allometric power, W/m² | Men | 1,542 | 196 | 0.879 | 0.275 |
|  | Women | 1,593 | 129 | 0.323 | 0.517 |
| Relative power, W/kg | Men | 1,542 | 196 | 0.960 | 0.282 |
|  | Women | 1,593 | 129 | 0.282 | 0.510 |
| Whole-body FMR | Men | 1,548 | 199 | 0.692 | 0.221 |
|  | Women | 1,620 | 131 | 0.717 | 0.582 |
| Trunk FMR | Men | 1,548 | 199 | 0.798 | 0.229 |
|  | Women | 1,620 | 131 | 0.845 | 0.598 |
| Arm FMR | Men | 1,548 | 199 | 0.330 | 0.173 |
|  | Women | 1,620 | 131 | 0.648 | 0.572 |
| Leg FMR | Men | 1,548 | 199 | 0.798 | 0.229 |
|  | Women | 1,620 | 131 | 0.682 | 0.579 |
| Whole-body FMI | Men | 1,548 | 199 | 0.559 | 0.218 |
|  | Women | 1,620 | 131 | 0.815 | 0.601 |
| Trunk FMI | Men | 1,548 | 199 | 0.525 | 0.217 |
|  | Women | 1,620 | 131 | 0.828 | 0.604 |
| Arm FMI | Men | 1,548 | 199 | 0.479 | 0.208 |
|  | Women | 1,620 | 131 | 0.807 | 0.599 |
| Leg FMI | Men | 1,548 | 199 | 0.786 | 0.229 |
|  | Women | 1,620 | 131 | 0.796 | 0.596 |
| Trunk-to-total fat ratio | Men | 1,548 | 199 | 0.864 | 0.239 |
|  | Women | 1,620 | 131 | 0.746 | 0.611 |
| Arm-to-total fat ratio | Men | 1,548 | 199 | 0.737 | 0.226 |
|  | Women | 1,620 | 131 | 0.889 | 0.610 |
| Leg-to-total fat ratio | Men | 1,548 | 199 | 0.762 | 0.236 |
|  | Women | 1,620 | 131 | 0.915 | 0.614 |
| **Coronary heart disease** |  |  |  |  |  |
| PBF, % | Men | 1,683 | 113 | 0.805 | 0.688 |
|  | Women | 1,725 | 64 | 0.449 | 0.504 |
| FMI, kg/m² | Men | 1,683 | 113 | 0.913 | 0.694 |
|  | Women | 1,725 | 64 | 0.286 | 0.454 |
| ALM/weight, % | Men | 1,683 | 113 | 0.175 | 0.552 |
|  | Women | 1,725 | 64 | 0.776 | 0.621 |
| ALM/height², kg/m² | Men | 1,683 | 113 | 0.180 | 0.586 |
|  | Women | 1,725 | 64 | 0.243 | 0.490 |
| BMI, kg/m² | Men | 1,683 | 113 | 0.715 | 0.693 |
|  | Women | 1,725 | 64 | 0.233 | 0.450 |
| WC, cm | Men | 1,683 | 113 | 0.893 | 0.690 |
|  | Women | 1,724 | 64 | 0.588 | 0.643 |
| Grip strength, kg | Men | 1,683 | 113 | 0.100 | 0.492 |
|  | Women | 1,725 | 64 | 0.925 | 0.628 |
| Gait speed, m/s | Men | 1,683 | 113 | 0.829 | 0.701 |
|  | Women | 1,725 | 64 | 0.682 | 0.589 |
| Five-times chair stand, s | Men | 1,674 | 111 | 0.157 | 0.549 |
|  | Women | 1,696 | 62 | 0.192 | 0.424 |
| Absolute power, W | Men | 1,674 | 111 | 0.887 | 0.733 |
|  | Women | 1,696 | 62 | 0.674 | 0.499 |
| Allometric power, W/m² | Men | 1,674 | 111 | 0.610 | 0.707 |
|  | Women | 1,696 | 62 | 0.630 | 0.496 |
| Relative power, W/kg | Men | 1,674 | 111 | 0.508 | 0.701 |
|  | Women | 1,696 | 62 | 0.189 | 0.405 |
| Whole-body FMR | Men | 1,683 | 113 | 0.829 | 0.690 |
|  | Women | 1,725 | 64 | 0.460 | 0.517 |
| Trunk FMR | Men | 1,683 | 113 | 0.908 | 0.689 |
|  | Women | 1,725 | 64 | 0.588 | 0.573 |
| Arm FMR | Men | 1,683 | 113 | 0.766 | 0.692 |
|  | Women | 1,725 | 64 | 0.272 | 0.459 |
| Leg FMR | Men | 1,683 | 113 | 0.820 | 0.695 |
|  | Women | 1,725 | 64 | 0.603 | 0.571 |
| Whole-body FMI | Men | 1,683 | 113 | 0.913 | 0.694 |
|  | Women | 1,725 | 64 | 0.286 | 0.454 |
| Trunk FMI | Men | 1,683 | 113 | 0.995 | 0.695 |
|  | Women | 1,725 | 64 | 0.352 | 0.511 |
| Arm FMI | Men | 1,683 | 113 | 0.628 | 0.680 |
|  | Women | 1,725 | 64 | 0.198 | 0.412 |
| Leg FMI | Men | 1,683 | 113 | 0.865 | 0.686 |
|  | Women | 1,725 | 64 | 0.360 | 0.485 |
| Trunk-to-total fat ratio | Men | 1,683 | 113 | 0.808 | 0.674 |
|  | Women | 1,725 | 64 | 0.893 | 0.647 |
| Arm-to-total fat ratio | Men | 1,683 | 113 | 0.253 | 0.611 |
|  | Women | 1,725 | 64 | 0.545 | 0.616 |
| Leg-to-total fat ratio | Men | 1,683 | 113 | 0.962 | 0.696 |
|  | Women | 1,725 | 64 | 0.725 | 0.625 |
| **Stroke** |  |  |  |  |  |
| PBF, % | Men | 1,891 | 94 | 0.941 | 0.323 |
|  | Women | 1,934 | 56 | 0.998 | 0.431 |
| FMI, kg/m² | Men | 1,891 | 94 | 0.327 | 0.284 |
|  | Women | 1,934 | 56 | 0.743 | 0.427 |
| ALM/weight, % | Men | 1,891 | 94 | 0.627 | 0.315 |
|  | Women | 1,934 | 56 | 0.401 | 0.375 |
| ALM/height², kg/m² | Men | 1,891 | 94 | 0.300 | 0.286 |
|  | Women | 1,934 | 56 | 0.136 | 0.273 |
| BMI, kg/m² | Men | 1,891 | 94 | 0.148 | 0.225 |
|  | Women | 1,934 | 56 | 0.595 | 0.414 |
| WC, cm | Men | 1,891 | 94 | 0.872 | 0.324 |
|  | Women | 1,933 | 56 | 0.509 | 0.393 |
| Grip strength, kg | Men | 1,891 | 94 | 0.455 | 0.322 |
|  | Women | 1,934 | 56 | 0.404 | 0.367 |
| Gait speed, m/s | Men | 1,891 | 94 | 0.725 | 0.328 |
|  | Women | 1,934 | 56 | 0.546 | 0.422 |
| Five-times chair stand, s | Men | 1,880 | 92 | 0.790 | 0.260 |
|  | Women | 1,903 | 56 | 0.484 | 0.400 |
| Absolute power, W | Men | 1,880 | 92 | 0.177 | 0.211 |
|  | Women | 1,903 | 56 | 0.559 | 0.415 |
| Allometric power, W/m² | Men | 1,880 | 92 | 0.124 | 0.188 |
|  | Women | 1,903 | 56 | 0.470 | 0.404 |
| Relative power, W/kg | Men | 1,880 | 92 | 0.328 | 0.238 |
|  | Women | 1,903 | 56 | 0.700 | 0.428 |
| Whole-body FMR | Men | 1,891 | 94 | 0.913 | 0.326 |
|  | Women | 1,934 | 56 | 0.948 | 0.430 |
| Trunk FMR | Men | 1,891 | 94 | 0.954 | 0.326 |
|  | Women | 1,934 | 56 | 0.732 | 0.416 |
| Arm FMR | Men | 1,891 | 94 | 0.961 | 0.327 |
|  | Women | 1,934 | 56 | 0.908 | 0.431 |
| Leg FMR | Men | 1,891 | 94 | 0.942 | 0.322 |
|  | Women | 1,934 | 56 | 0.809 | 0.429 |
| Whole-body FMI | Men | 1,891 | 94 | 0.327 | 0.284 |
|  | Women | 1,934 | 56 | 0.743 | 0.427 |
| Trunk FMI | Men | 1,891 | 94 | 0.298 | 0.282 |
|  | Women | 1,934 | 56 | 0.983 | 0.431 |
| Arm FMI | Men | 1,891 | 94 | 0.657 | 0.325 |
|  | Women | 1,934 | 56 | 0.712 | 0.429 |
| Leg FMI | Men | 1,891 | 94 | 0.432 | 0.289 |
|  | Women | 1,934 | 56 | 0.478 | 0.401 |
| Trunk-to-total fat ratio | Men | 1,891 | 94 | 0.987 | 0.341 |
|  | Women | 1,934 | 56 | 0.219 | 0.331 |
| Arm-to-total fat ratio | Men | 1,891 | 94 | 0.193 | 0.233 |
|  | Women | 1,934 | 56 | 0.590 | 0.426 |
| Leg-to-total fat ratio | Men | 1,891 | 94 | 0.971 | 0.334 |
|  | Women | 1,934 | 56 | 0.274 | 0.340 |
| **Heart failure** |  |  |  |  |  |
| PBF, % | Men | 1,927 | 77 | 0.212 | 0.467 |
|  | Women | 1,922 | 53 | 0.123 | 0.323 |
| FMI, kg/m² | Men | 1,927 | 77 | 0.330 | 0.564 |
|  | Women | 1,922 | 53 | 0.110 | 0.320 |
| ALM/weight, % | Men | 1,927 | 77 | 0.125 | 0.318 |
|  | Women | 1,922 | 53 | 0.276 | 0.448 |
| ALM/height², kg/m² | Men | 1,927 | 77 | 0.678 | 0.506 |
|  | Women | 1,922 | 53 | 0.483 | 0.486 |
| BMI, kg/m² | Men | 1,927 | 77 | 0.619 | 0.623 |
|  | Women | 1,922 | 53 | 0.178 | 0.381 |
| WC, cm | Men | 1,927 | 77 | 0.791 | 0.627 |
|  | Women | 1,921 | 53 | 0.525 | 0.513 |
| Grip strength, kg | Men | 1,927 | 77 | 0.785 | 0.488 |
|  | Women | 1,922 | 53 | 0.452 | 0.493 |
| Gait speed, m/s | Men | 1,927 | 77 | 0.690 | 0.612 |
|  | Women | 1,922 | 53 | 0.491 | 0.539 |
| Five-times chair stand, s | Men | 1,915 | 76 | 0.515 | 0.621 |
|  | Women | 1,889 | 51 | 0.205 | 0.305 |
| Absolute power, W | Men | 1,915 | 76 | 0.343 | 0.626 |
|  | Women | 1,889 | 51 | 0.466 | 0.478 |
| Allometric power, W/m² | Men | 1,915 | 76 | 0.434 | 0.631 |
|  | Women | 1,889 | 51 | 0.487 | 0.488 |
| Relative power, W/kg | Men | 1,915 | 76 | 0.445 | 0.625 |
|  | Women | 1,889 | 51 | 0.198 | 0.370 |
| Whole-body FMR | Men | 1,927 | 77 | 0.180 | 0.437 |
|  | Women | 1,922 | 53 | 0.108 | 0.309 |
| Trunk FMR | Men | 1,927 | 77 | 0.237 | 0.495 |
|  | Women | 1,922 | 53 | 0.210 | 0.393 |
| Arm FMR | Men | 1,927 | 77 | 0.052 | 0.244 |
|  | Women | 1,922 | 53 | 0.207 | 0.381 |
| Leg FMR | Men | 1,927 | 77 | 0.334 | 0.493 |
|  | Women | 1,922 | 53 | 0.103 | 0.321 |
| Whole-body FMI | Men | 1,927 | 77 | 0.330 | 0.564 |
|  | Women | 1,922 | 53 | 0.110 | 0.320 |
| Trunk FMI | Men | 1,927 | 77 | 0.312 | 0.564 |
|  | Women | 1,922 | 53 | 0.173 | 0.380 |
| Arm FMI | Men | 1,927 | 77 | 0.170 | 0.468 |
|  | Women | 1,922 | 53 | 0.143 | 0.341 |
| Leg FMI | Men | 1,927 | 77 | 0.603 | 0.602 |
|  | Women | 1,922 | 53 | 0.089 | 0.301 |
| Trunk-to-total fat ratio | Men | 1,927 | 77 | 0.341 | 0.555 |
|  | Women | 1,922 | 53 | 0.843 | 0.553 |
| Arm-to-total fat ratio | Men | 1,927 | 77 | 0.184 | 0.448 |
|  | Women | 1,922 | 53 | 0.631 | 0.529 |
| Leg-to-total fat ratio | Men | 1,927 | 77 | 0.362 | 0.551 |
|  | Women | 1,922 | 53 | 0.865 | 0.549 |

Tests were based on scaled Schoenfeld residuals from the fully adjusted models (Model 2) used in Tables 2 and 3 of the main text and in Supplementary Table S9-11. A P value above 0.05 is consistent with the proportional-hazards assumption. No evidence of PH violation was found for any exposure. Statistical power to detect departures is limited for the less common outcomes.

**Supplementary Table S8** Sex differences in regional fat distribution.

| **Characteristic** | **Male (n = 1548)** | **Female (n = 1620)** | **P value** |
| --- | --- | --- | --- |
| ***Fat-to-muscle ratio (FMR)*** | | | |
| Whole-body FMR | 0.34 (0.28-0.40) | 0.56 (0.48-0.64) | <0.001 |
| Trunk FMR | 0.40 (0.32-0.47) | 0.58 (0.49-0.67) | <0.001 |
| Arm FMR | 0.32 (0.26-0.39) | 0.70 (0.58-0.85) | <0.001 |
| Leg FMR | 0.28 (0.23-0.33) | 0.55 (0.45-0.66) | <0.001 |
| ***Regional fat mass index, kg/m²*** | | | |
| Whole-body fat mass index | 5.59 (4.59-6.65) | 8.18 (6.84-9.63) | <0.001 |
| Trunk fat mass index | 3.20 (2.49-3.89) | 4.30 (3.48-5.08) | <0.001 |
| Arm fat mass index | 0.60 (0.48-0.74) | 1.06 (0.84-1.30) | <0.001 |
| Leg fat mass index | 1.46 (1.20-1.78) | 2.45 (1.97-3.01) | <0.001 |
| ***Regional-to-total fat ratio*** | | | |
| Trunk-to-total fat ratio | 0.564 (0.524-0.595) | 0.518 (0.484-0.552) | <0.001 |
| Arm-to-total fat ratio | 0.108 (0.100-0.117) | 0.129 (0.118-0.140) | <0.001 |
| Leg-to-total fat ratio | 0.266 (0.240-0.297) | 0.306 (0.271-0.341) | <0.001 |

Data were median (interquartile range). P values were derived from the Mann-Whitney U test comparing men versus women. FMR was calculated as regional fat mass divided by regional lean mass; regional fat mass index was calculated as regional fat mass divided by height squared; regional-to-total fat ratio was calculated as regional fat mass divided by whole-body fat mass. FMR, fat-to-muscle ratio.

**Supplementary Table S9** Sensitivity analysis adding CVD deaths (without prior CVD hospitalization) to the incident CVD outcome definition (Cox model).

|  | **Total (n = 3168)** | | **Male (n = 1548)** | | **Female (n = 1620)** | | **P for interaction** |
| --- | --- | --- | --- | --- | --- | --- | --- |
| **Characteristic** | **HR (95% CI)** | **P** | **HR (95% CI)** | **P** | **HR (95% CI)** | **P** |  |
| No. of incident CVD | 351 | | 217 | | 134 | |  |
| **Model 1** |  |  |  |  |  |  |  |
| ***Body composition*** |  |  |  |  |  |  |  |
| PBF, % | 1.16 (1.00-1.35) | 0.056 | 1.24 (1.08-1.42) | 0.003 | 0.95 (0.81-1.12) | 0.566 | 0.014 |
| FMI, kg/m² | 1.18 (1.04-1.34) | 0.011 | 1.25 (1.10-1.43) | <0.001 | 1.03 (0.87-1.22) | 0.764 | 0.029 |
| ALM/weight, % | 0.81 (0.69-0.95) | 0.009 | 0.79 (0.69-0.91) | <0.001 | 0.99 (0.84-1.17) | 0.909 | 0.037 |
| ALM/height², kg/m² | 1.12 (0.98-1.27) | 0.094 | 1.11 (0.96-1.27) | 0.152 | 1.08 (0.91-1.28) | 0.370 | 0.983 |
| BMI, kg/m² | 1.17 (1.05-1.30) | 0.003 | 1.23 (1.08-1.41) | 0.002 | 1.08 (0.92-1.28) | 0.350 | 0.193 |
| WC, cm | 1.21 (1.09-1.35) | <0.001 | 1.29 (1.13-1.48) | <0.001 | 1.11 (0.94-1.30) | 0.229 | 0.115 |
| ***Muscle strength and function*** | | | | | | | |
| Grip strength, kg | 0.79 (0.68-0.92) | 0.003 | 0.84 (0.73-0.97) | 0.016 | 0.84 (0.70-1.01) | 0.058 | 0.559 |
| Gait speed, m/s | 0.87 (0.78-0.97) | 0.016 | 0.90 (0.78-1.04) | 0.145 | 0.83 (0.70-0.99) | 0.040 | 0.553 |
| Five-times chair stand, s | 1.10 (1.00-1.21) | 0.052 | 1.05 (0.92-1.20) | 0.482 | 1.14 (1.00-1.30) | 0.047 | 0.572 |
| ***Muscle power*** | | | | | | | |
| Absolute power, W | 0.96 (0.85-1.08) | 0.506 | 1.03 (0.90-1.19) | 0.658 | 0.81 (0.66-0.98) | 0.035 | 0.046 |
| Allometric power, W/m² | 0.96 (0.86-1.08) | 0.521 | 1.04 (0.90-1.19) | 0.588 | 0.82 (0.68-1.00) | 0.051 | 0.062 |
| Relative power, W/kg | 0.88 (0.78-0.99) | 0.037 | 0.95 (0.82-1.09) | 0.462 | 0.77 (0.64-0.93) | 0.008 | 0.073 |
| **Model 2** | | | | | | | |
| ***Body composition*** | | | | | | | |
| PBF, % | 1.07 (0.92-1.25) | 0.382 | 1.18 (1.02-1.36) | 0.025 | 0.90 (0.76-1.07) | 0.247 | 0.033 |
| FMI, kg/m² | 1.09 (0.96-1.25) | 0.189 | 1.19 (1.03-1.36) | 0.016 | 0.96 (0.81-1.15) | 0.657 | 0.072 |
| ALM/weight, % | 0.89 (0.76-1.05) | 0.168 | 0.84 (0.73-0.97) | 0.020 | 1.05 (0.89-1.25) | 0.562 | 0.084 |
| ALM/height², kg/m² | 1.06 (0.93-1.21) | 0.396 | 1.06 (0.93-1.23) | 0.377 | 1.02 (0.86-1.22) | 0.793 | 0.868 |
| BMI, kg/m² | 1.09 (0.98-1.22) | 0.120 | 1.16 (1.01-1.33) | 0.037 | 1.00 (0.84-1.20) | 0.988 | 0.262 |
| WC, cm | 1.13 (1.01-1.27) | 0.026 | 1.23 (1.06-1.42) | 0.005 | 1.02 (0.86-1.22) | 0.790 | 0.156 |
| ***Muscle strength and function*** | | | | | | | |
| Grip strength, kg | 0.79 (0.68-0.92) | 0.002 | 0.84 (0.72-0.97) | 0.020 | 0.83 (0.69-0.99) | 0.044 | 0.445 |
| Gait speed, m/s | 0.89 (0.79-1.00) | 0.051 | 0.92 (0.79-1.07) | 0.276 | 0.84 (0.70-1.02) | 0.074 | 0.471 |
| Five-times chair stand, s | 1.09 (0.99-1.21) | 0.073 | 1.04 (0.91-1.19) | 0.539 | 1.14 (0.99-1.31) | 0.060 | 0.468 |
| ***Muscle power*** | | | | | | | |
| Absolute power, W | 0.93 (0.82-1.06) | 0.281 | 1.01 (0.87-1.16) | 0.917 | 0.77 (0.63-0.95) | 0.014 | 0.033 |
| Allometric power, W/m² | 0.94 (0.83-1.05) | 0.274 | 1.01 (0.88-1.17) | 0.878 | 0.79 (0.65-0.97) | 0.024 | 0.054 |
| Relative power, W/kg | 0.89 (0.79-1.00) | 0.053 | 0.95 (0.82-1.10) | 0.501 | 0.77 (0.64-0.94) | 0.010 | 0.066 |

Values were cause-specific hazard ratios (95% CI) per 1-SD increase, estimated from Cox proportional-hazards models. Model 1 was adjusted for age in the sex-stratified analyses and additionally adjusted for sex in the total population. Model 2 was additionally adjusted for education level, smoking, alcohol drinking, physical activity, energy intake, diabetes, hypertension, cancer history and lipid-lowering medication. HR, hazard ratio; CI, confidence interval; CVD, cardiovascular disease; PBF, percentage of body fat; FMI, fat mass index; ALM, appendicular lean mass; BMI, body mass index; WC, waist circumference.

**Supplementary Table S10** Sensitivity analysis adding CVD deaths (without prior CVD hospitalization) to the incident CVD outcome definition (Fine-gray model).

|  | **Total (n = 3168)** | | **Male (n = 1548)** | | **Female (n = 1620)** | |
| --- | --- | --- | --- | --- | --- | --- |
| **Characteristic** | **HR (95% CI)** | **P** | **HR (95% CI)** | **P** | **HR (95% CI)** | **P** |
| No. of incident CVD | 351 | | 217 | | 134 | |
| **Model 1** |  |  |  |  |  |  |
| ***Body composition*** |  |  |  |  |  |  |
| PBF, % | 1.16 (0.99-1.36) | 0.057 | 1.25 (1.09-1.43) | 0.002 | 0.95 (0.81-1.13) | 0.594 |
| FMI, kg/m² | 1.18 (1.04-1.34) | 0.011 | 1.26 (1.10-1.43) | <0.001 | 1.02 (0.86-1.21) | 0.792 |
| ALM/weight, % | 0.81 (0.69-0.95) | 0.010 | 0.79 (0.69-0.91) | <0.001 | 0.99 (0.83-1.18) | 0.896 |
| ALM/height², kg/m² | 1.12 (0.98-1.28) | 0.102 | 1.11 (0.96-1.28) | 0.160 | 1.08 (0.91-1.27) | 0.385 |
| BMI, kg/m² | 1.17 (1.05-1.30) | 0.003 | 1.24 (1.08-1.42) | 0.002 | 1.08 (0.92-1.27) | 0.364 |
| WC, cm | 1.21 (1.08-1.34) | <0.001 | 1.29 (1.12-1.49) | <0.001 | 1.10 (0.94-1.29) | 0.252 |
| ***Muscle strength and function*** |  |  |  |  |  |  |
| Grip strength, kg | 0.80 (0.69-0.93) | 0.003 | 0.85 (0.73-0.98) | 0.022 | 0.84 (0.70-1.00) | 0.048 |
| Gait speed, m/s | 0.88 (0.78-0.98) | 0.024 | 0.91 (0.78-1.05) | 0.188 | 0.84 (0.70-0.99) | 0.042 |
| Five-times chair stand, s | 1.10 (1.00-1.21) | 0.053 | 1.05 (0.91-1.21) | 0.536 | 1.14 (1.01-1.28) | 0.030 |
| ***Muscle power*** |  |  |  |  |  |  |
| Absolute power, W | 0.96 (0.85-1.09) | 0.548 | 1.04 (0.91-1.19) | 0.591 | 0.81 (0.66-1.00) | 0.046 |
| Allometric power, W/m² | 0.97 (0.86-1.08) | 0.561 | 1.04 (0.92-1.19) | 0.513 | 0.82 (0.67-1.01) | 0.062 |
| Relative power, W/kg | 0.88 (0.79-0.99) | 0.041 | 0.95 (0.83-1.09) | 0.480 | 0.78 (0.64-0.93) | 0.007 |
| **Model 2** |  |  |  |  |  |  |
| ***Body composition*** |  |  |  |  |  |  |
| PBF, % | 1.07 (0.91-1.26) | 0.391 | 1.19 (1.03-1.37) | 0.020 | 0.90 (0.75-1.08) | 0.265 |
| FMI, kg/m² | 1.09 (0.95-1.25) | 0.203 | 1.19 (1.04-1.37) | 0.013 | 0.95 (0.80-1.15) | 0.614 |
| ALM/weight, % | 0.90 (0.76-1.06) | 0.193 | 0.84 (0.73-0.97) | 0.019 | 1.05 (0.88-1.26) | 0.566 |
| ALM/height², kg/m² | 1.06 (0.93-1.22) | 0.390 | 1.07 (0.93-1.24) | 0.355 | 1.02 (0.86-1.21) | 0.833 |
| BMI, kg/m² | 1.09 (0.97-1.22) | 0.132 | 1.16 (1.01-1.34) | 0.037 | 0.99 (0.83-1.19) | 0.946 |
| WC, cm | 1.13 (1.00-1.26) | 0.043 | 1.22 (1.05-1.42) | 0.009 | 1.01 (0.85-1.21) | 0.892 |
| ***Muscle strength and function*** |  |  |  |  |  |  |
| Grip strength, kg | 0.80 (0.68-0.93) | 0.004 | 0.85 (0.73-0.98) | 0.028 | 0.83 (0.69-0.99) | 0.038 |
| Gait speed, m/s | 0.89 (0.79-1.01) | 0.063 | 0.92 (0.79-1.08) | 0.309 | 0.85 (0.71-1.02) | 0.089 |
| Five-times chair stand, s | 1.09 (0.99-1.21) | 0.072 | 1.04 (0.90-1.20) | 0.575 | 1.14 (1.00-1.29) | 0.046 |
| ***Muscle power*** |  |  |  |  |  |  |
| Absolute power, W | 0.94 (0.82-1.06) | 0.297 | 1.01 (0.88-1.16) | 0.877 | 0.77 (0.62-0.96) | 0.021 |
| Allometric power, W/m² | 0.94 (0.83-1.05) | 0.285 | 1.01 (0.89-1.16) | 0.824 | 0.79 (0.64-0.98) | 0.032 |
| Relative power, W/kg | 0.89 (0.79-1.00) | 0.053 | 0.95 (0.83-1.10) | 0.501 | 0.78 (0.64-0.94) | 0.009 |

Values were subdistribution hazard ratios (95% CI) per 1-SD increase, estimated from Fine-Gray models with non-CVD death as the competing event. Model 1 was adjusted for age in the sex-stratified analyses and additionally adjusted for sex in the total population. Model 2 was additionally adjusted for education level, smoking, alcohol drinking, physical activity, energy intake, diabetes, hypertension, cancer history and lipid-lowering medication use. Abbreviations: HR, hazard ratio; CI, confidence interval; CVD, cardiovascular disease; PBF, percentage of body fat; FMI, fat mass index; ALM, appendicular lean mass; BMI, body mass index; WC, waist circumference.

**Supplementary Table S11.** Complete-case analysis restricted to participants with complete data on all exposures, covariates, and outcomes (n = 3,130).

|  | **Total (n = 3,130)** | | **Male (n = 1,541)** | | **Female (n = 1,589)** | | **P for**  **interaction** |
| --- | --- | --- | --- | --- | --- | --- | --- |
| **Characteristic** | **HR (95% CI)** | **P** | **HR (95% CI)** | **P** | **HR (95% CI)** | **P** |  |
| No. of incident CVD | 325 | | 196 | | 129 | |  |
| **Model 1** |  |  |  |  |  |  |  |
| ***Body composition*** |  |  |  |  |  |  |  |
| PBF, % | 1.17 (1.00-1.37) | 0.054 | 1.29 (1.11-1.49) | <0.001 | 0.93 (0.79-1.09) | 0.378 | 0.003 |
| FMI, kg/m² | 1.19 (1.04-1.36) | 0.009 | 1.31 (1.14-1.51) | <0.001 | 1.00 (0.84-1.19) | 0.982 | 0.005 |
| ALM/weight, % | 0.80 (0.68-0.94) | 0.007 | 0.76 (0.66-0.88) | <0.001 | 1.01 (0.85-1.19) | 0.922 | 0.011 |
| ALM/height², kg/m² | 1.14 (0.99-1.30) | 0.066 | 1.13 (0.98-1.31) | 0.094 | 1.07 (0.90-1.28) | 0.414 | 0.783 |
| BMI, kg/m² | 1.19 (1.07-1.33) | 0.002 | 1.29 (1.12-1.48) | <0.001 | 1.07 (0.90-1.27) | 0.466 | 0.068 |
| WC, cm | 1.21 (1.09-1.35) | <0.001 | 1.31 (1.14-1.51) | <0.001 | 1.10 (0.93-1.30) | 0.276 | 0.085 |
| ***Muscle strength and function*** |  |  |  |  |  |  |  |
| Grip strength, kg | 0.79 (0.68-0.93) | 0.004 | 0.84 (0.72-0.98) | 0.022 | 0.84 (0.69-1.01) | 0.064 | 0.528 |
| Gait speed, m/s | 0.86 (0.76-0.97) | 0.011 | 0.89 (0.77-1.03) | 0.124 | 0.82 (0.69-0.98) | 0.031 | 0.506 |
| Five-times chair stand, s | 1.11 (1.01-1.22) | 0.037 | 1.06 (0.93-1.22) | 0.389 | 1.14 (1.00-1.30) | 0.043 | 0.671 |
| ***Muscle power*** |  |  |  |  |  |  |  |
| Absolute power, W | 0.94 (0.83-1.07) | 0.364 | 1.02 (0.88-1.18) | 0.834 | 0.80 (0.65-0.98) | 0.032 | 0.059 |
| Allometric power, W/m² | 0.95 (0.84-1.07) | 0.408 | 1.03 (0.89-1.19) | 0.704 | 0.82 (0.67-1.00) | 0.046 | 0.073 |
| Relative power, W/kg | 0.86 (0.76-0.97) | 0.018 | 0.92 (0.79-1.07) | 0.285 | 0.77 (0.64-0.94) | 0.009 | 0.127 |
| ***Fat-to-muscle ratio (FMR)*** |  |  |  |  |  |  |  |
| Whole-body FMR | 1.14 (0.97-1.33) | 0.105 | 1.27 (1.11-1.46) | <0.001 | 0.94 (0.80-1.12) | 0.492 | 0.002 |
| Trunk FMR | 1.15 (1.01-1.32) | 0.041 | 1.30 (1.13-1.50) | <0.001 | 0.95 (0.80-1.12) | 0.519 | 0.002 |
| Arm FMR | 1.17 (0.99-1.38) | 0.071 | 1.24 (1.09-1.41) | 0.001 | 1.03 (0.87-1.21) | 0.770 | 0.010 |
| Leg FMR | 1.01 (0.85-1.20) | 0.908 | 1.13 (0.98-1.29) | 0.083 | 0.92 (0.78-1.09) | 0.348 | 0.050 |
| ***Regional fat mass index (FMI)*** |  |  |  |  |  |  |  |
| Whole-body FMI | 1.19 (1.04-1.36) | 0.009 | 1.31 (1.14-1.51) | <0.001 | 1.00 (0.84-1.19) | 0.982 | 0.005 |
| Trunk FMI | 1.21 (1.07-1.37) | 0.002 | 1.35 (1.18-1.56) | <0.001 | 1.01 (0.85-1.20) | 0.923 | 0.004 |
| Arm FMI | 1.17 (1.01-1.35) | 0.032 | 1.23 (1.08-1.41) | 0.002 | 1.04 (0.87-1.23) | 0.673 | 0.025 |
| Leg FMI | 1.08 (0.93-1.25) | 0.297 | 1.17 (1.03-1.34) | 0.020 | 0.97 (0.81-1.15) | 0.695 | 0.039 |
| ***Regional-to-total fat ratio*** |  |  |  |  |  |  |  |
| Trunk-to-total fat ratio | 1.18 (1.05-1.33) | 0.005 | 1.31 (1.13-1.53) | <0.001 | 1.00 (0.85-1.19) | 0.965 | 0.024 |
| Arm-to-total fat ratio | 1.03 (0.90-1.18) | 0.648 | 0.98 (0.85-1.12) | 0.726 | 1.08 (0.91-1.28) | 0.379 | 0.394 |
| Leg-to-total fat ratio | 0.86 (0.76-0.97) | 0.012 | 0.81 (0.70-0.94) | 0.005 | 0.94 (0.79-1.11) | 0.478 | 0.121 |
| **Model 2** |  |  |  |  |  |  |  |
| ***Body composition*** |  |  |  |  |  |  |  |
| PBF, % | 1.09 (0.93-1.29) | 0.281 | 1.24 (1.07-1.45) | 0.005 | 0.88 (0.74-1.05) | 0.154 | 0.007 |
| FMI, kg/m² | 1.12 (0.97-1.28) | 0.117 | 1.25 (1.09-1.45) | 0.002 | 0.94 (0.78-1.12) | 0.494 | 0.014 |
| ALM/weight, % | 0.86 (0.73-1.02) | 0.094 | 0.79 (0.68-0.93) | 0.003 | 1.07 (0.90-1.28) | 0.447 | 0.025 |
| ALM/height², kg/m² | 1.08 (0.94-1.24) | 0.297 | 1.09 (0.94-1.26) | 0.255 | 1.02 (0.85-1.22) | 0.846 | 0.696 |
| BMI, kg/m² | 1.12 (1.00-1.25) | 0.059 | 1.22 (1.06-1.41) | 0.007 | 0.99 (0.82-1.18) | 0.872 | 0.098 |
| WC, cm | 1.14 (1.02-1.28) | 0.023 | 1.25 (1.08-1.45) | 0.003 | 1.02 (0.85-1.21) | 0.855 | 0.118 |
| ***Muscle strength and function*** |  |  |  |  |  |  |  |
| Grip strength, kg | 0.78 (0.67-0.92) | 0.003 | 0.83 (0.72-0.97) | 0.022 | 0.82 (0.67-0.99) | 0.041 | 0.386 |
| Gait speed, m/s | 0.87 (0.77-0.99) | 0.032 | 0.90 (0.77-1.05) | 0.192 | 0.84 (0.70-1.01) | 0.068 | 0.451 |
| Five-times chair stand, s | 1.10 (1.00-1.22) | 0.051 | 1.06 (0.92-1.21) | 0.434 | 1.15 (1.00-1.32) | 0.056 | 0.550 |
| ***Muscle power*** |  |  |  |  |  |  |  |
| Absolute power, W | 0.92 (0.80-1.05) | 0.195 | 0.99 (0.85-1.15) | 0.912 | 0.77 (0.62-0.95) | 0.013 | 0.044 |
| Allometric power, W/m² | 0.92 (0.82-1.05) | 0.214 | 1.00 (0.86-1.16) | 0.981 | 0.79 (0.64-0.97) | 0.022 | 0.064 |
| Relative power, W/kg | 0.87 (0.76-0.98) | 0.026 | 0.92 (0.79-1.08) | 0.304 | 0.77 (0.64-0.94) | 0.011 | 0.115 |
| ***Fat-to-muscle ratio (FMR)*** |  |  |  |  |  |  |  |
| Whole-body FMR | 1.07 (0.91-1.26) | 0.401 | 1.23 (1.06-1.42) | 0.006 | 0.90 (0.75-1.07) | 0.245 | 0.009 |
| Trunk FMR | 1.07 (0.93-1.24) | 0.320 | 1.25 (1.08-1.45) | 0.004 | 0.88 (0.74-1.05) | 0.171 | 0.005 |
| Arm FMR | 1.10 (0.92-1.31) | 0.304 | 1.19 (1.04-1.37) | 0.011 | 0.97 (0.81-1.16) | 0.757 | 0.039 |
| Leg FMR | 1.01 (0.85-1.21) | 0.874 | 1.12 (0.97-1.28) | 0.122 | 0.93 (0.78-1.12) | 0.455 | 0.110 |
| ***Regional fat mass index (FMI)*** |  |  |  |  |  |  |  |
| Whole-body FMI | 1.12 (0.97-1.28) | 0.117 | 1.25 (1.09-1.45) | 0.002 | 0.94 (0.78-1.12) | 0.494 | 0.014 |
| Trunk FMI | 1.12 (0.99-1.28) | 0.072 | 1.29 (1.11-1.50) | <0.001 | 0.92 (0.77-1.10) | 0.378 | 0.008 |
| Arm FMI | 1.08 (0.93-1.26) | 0.284 | 1.18 (1.03-1.35) | 0.018 | 0.96 (0.80-1.15) | 0.658 | 0.062 |
| Leg FMI | 1.07 (0.92-1.23) | 0.394 | 1.15 (1.00-1.32) | 0.045 | 0.97 (0.81-1.15) | 0.697 | 0.085 |
| ***Regional-to-total fat ratio*** |  |  |  |  |  |  |  |
| Trunk-to-total fat ratio | 1.08 (0.96-1.22) | 0.211 | 1.23 (1.05-1.45) | 0.010 | 0.91 (0.76-1.08) | 0.267 | 0.026 |
| Arm-to-total fat ratio | 0.98 (0.86-1.13) | 0.805 | 0.96 (0.83-1.11) | 0.605 | 0.99 (0.83-1.18) | 0.942 | 0.561 |
| Leg-to-total fat ratio | 0.94 (0.83-1.07) | 0.341 | 0.86 (0.74-1.01) | 0.061 | 1.05 (0.88-1.25) | 0.587 | 0.152 |

Of 3,168 participants, 3,130 had complete data and were included; 38 were excluded because of missing or imputed values. Values were hazard ratios (95% CI) per 1-SD increase from Cox proportional-hazards models. Model 1 was adjusted for age in the sex-stratified analyses and additionally adjusted for sex in the total population. Model 2 was additionally adjusted for education level, smoking, alcohol drinking, physical activity, energy intake, diabetes, hypertension, cancer history, and lipid-lowering medication. P values for interaction were derived from models containing the sex × exposure terms.

Abbreviations: ALM, appendicular lean mass; BMI, body mass index; CVD, cardiovascular disease; FMI, fat mass index; FMR, fat-to-muscle ratio; PASE, Physical Activity Scale for the Elderly; PBF, percentage of body fat; WC, waist circumference.

**Supplementary Table S12.** Sex-stratified associations of body composition, muscle strength, muscle power and regional fat distribution with incident CHD.

|  | **Total (n = 3,408)** | | **Male (n = 1,683)** | | **Female (n = 1,725)** | | **P for**  **interaction** |
| --- | --- | --- | --- | --- | --- | --- | --- |
| **Characteristic** | **HR (95% CI)** | **P** | **HR (95% CI)** | **P** | **HR (95% CI)** | **P** |  |
| No. of incident events | 177 | | 113 | | 64 | |  |
| **Model 1** |  |  |  |  |  |  |  |
| ***Body composition*** |  |  |  |  |  |  |  |
| PBF, % | 1.13 (0.91-1.40) | 0.260 | 1.29 (1.06-1.57) | 0.010 | 0.85 (0.67-1.06) | 0.154 | 0.006 |
| FMI, kg/m² | 1.15 (0.95-1.38) | 0.143 | 1.28 (1.07-1.54) | 0.007 | 0.92 (0.72-1.18) | 0.522 | 0.023 |
| ALM/weight, % | 0.88 (0.71-1.10) | 0.259 | 0.82 (0.68-0.99) | 0.039 | 1.11 (0.88-1.41) | 0.385 | 0.048 |
| ALM/height², kg/m² | 1.14 (0.95-1.37) | 0.152 | 1.14 (0.94-1.37) | 0.186 | 1.09 (0.85-1.39) | 0.491 | 0.965 |
| BMI, kg/m² | 1.14 (0.99-1.33) | 0.078 | 1.24 (1.03-1.49) | 0.023 | 1.01 (0.79-1.29) | 0.958 | 0.189 |
| WC, cm | 1.19 (1.03-1.38) | 0.020 | 1.32 (1.10-1.60) | 0.003 | 1.02 (0.80-1.30) | 0.878 | 0.077 |
| ***Muscle strength and function*** |  |  |  |  |  |  |  |
| Grip strength, kg | 0.87 (0.70-1.08) | 0.206 | 0.90 (0.74-1.11) | 0.327 | 0.91 (0.70-1.18) | 0.476 | 0.989 |
| Gait speed, m/s | 0.85 (0.73-1.00) | 0.050 | 0.91 (0.74-1.11) | 0.340 | 0.77 (0.60-0.99) | 0.043 | 0.447 |
| Five-times chair stand, s | 1.15 (1.02-1.31) | 0.023 | 1.04 (0.87-1.25) | 0.629 | 1.25 (1.07-1.47) | 0.006 | 0.292 |
| ***Muscle power*** |  |  |  |  |  |  |  |
| Absolute power, W | 0.92 (0.77-1.10) | 0.353 | 1.03 (0.85-1.25) | 0.788 | 0.69 (0.51-0.93) | 0.016 | 0.032 |
| Allometric power, W/m² | 0.91 (0.77-1.08) | 0.275 | 1.01 (0.83-1.23) | 0.891 | 0.72 (0.53-0.96) | 0.027 | 0.065 |
| Relative power, W/kg | 0.84 (0.71-0.99) | 0.041 | 0.91 (0.75-1.12) | 0.379 | 0.71 (0.53-0.94) | 0.015 | 0.130 |
| ***Fat-to-muscle ratio (FMR)*** |  |  |  |  |  |  |  |
| Whole-body FMR | 1.09 (0.88-1.35) | 0.448 | 1.26 (1.05-1.51) | 0.014 | 0.85 (0.67-1.09) | 0.203 | 0.008 |
| Trunk FMR | 1.16 (0.96-1.39) | 0.117 | 1.33 (1.10-1.60) | 0.003 | 0.90 (0.70-1.14) | 0.368 | 0.007 |
| Arm FMR | 1.01 (0.80-1.28) | 0.916 | 1.23 (1.03-1.46) | 0.021 | 0.84 (0.65-1.08) | 0.175 | 0.008 |
| Leg FMR | 0.92 (0.72-1.17) | 0.492 | 1.07 (0.89-1.28) | 0.452 | 0.84 (0.65-1.08) | 0.170 | 0.161 |
| ***Regional fat mass index (FMI)*** |  |  |  |  |  |  |  |
| Trunk FMI | 1.19 (1.01-1.41) | 0.036 | 1.34 (1.11-1.61) | 0.002 | 0.96 (0.75-1.22) | 0.717 | 0.025 |
| Arm FMI | 1.08 (0.88-1.31) | 0.467 | 1.22 (1.02-1.45) | 0.027 | 0.90 (0.70-1.15) | 0.394 | 0.029 |
| Leg FMI | 1.01 (0.82-1.23) | 0.934 | 1.12 (0.94-1.34) | 0.205 | 0.89 (0.69-1.15) | 0.364 | 0.132 |
| ***Regional-to-total fat ratio*** |  |  |  |  |  |  |  |
| Trunk-to-total fat ratio | 1.25 (1.06-1.47) | 0.007 | 1.40 (1.14-1.72) | 0.001 | 1.02 (0.80-1.30) | 0.880 | 0.061 |
| Arm-to-total fat ratio | 0.92 (0.77-1.11) | 0.394 | 0.98 (0.81-1.18) | 0.832 | 0.87 (0.68-1.11) | 0.272 | 0.585 |
| Leg-to-total fat ratio | 0.82 (0.70-0.97) | 0.022 | 0.76 (0.63-0.92) | 0.006 | 0.96 (0.75-1.22) | 0.714 | 0.107 |
| **Model 2** |  |  |  |  |  |  |  |
| ***Body composition*** |  |  |  |  |  |  |  |
| PBF, % | 1.03 (0.82-1.29) | 0.789 | 1.23 (1.00-1.50) | 0.049 | 0.80 (0.62-1.02) | 0.069 | 0.015 |
| FMI, kg/m² | 1.04 (0.86-1.27) | 0.660 | 1.21 (1.00-1.47) | 0.052 | 0.84 (0.65-1.10) | 0.213 | 0.059 |
| ALM/weight, % | 0.97 (0.77-1.22) | 0.812 | 0.87 (0.71-1.06) | 0.160 | 1.18 (0.91-1.51) | 0.206 | 0.089 |
| ALM/height², kg/m² | 1.05 (0.87-1.27) | 0.601 | 1.08 (0.89-1.31) | 0.427 | 0.98 (0.76-1.27) | 0.896 | 0.983 |
| BMI, kg/m² | 1.04 (0.89-1.22) | 0.590 | 1.16 (0.95-1.40) | 0.140 | 0.89 (0.69-1.17) | 0.412 | 0.292 |
| WC, cm | 1.09 (0.93-1.28) | 0.279 | 1.24 (1.02-1.51) | 0.033 | 0.91 (0.71-1.18) | 0.499 | 0.144 |
| ***Muscle strength and function*** |  |  |  |  |  |  |  |
| Grip strength, kg | 0.85 (0.68-1.06) | 0.148 | 0.90 (0.73-1.10) | 0.290 | 0.87 (0.66-1.14) | 0.312 | 0.867 |
| Gait speed, m/s | 0.86 (0.73-1.02) | 0.087 | 0.92 (0.75-1.14) | 0.465 | 0.77 (0.59-1.00) | 0.048 | 0.399 |
| Five-times chair stand, s | 1.15 (1.01-1.32) | 0.031 | 1.03 (0.86-1.23) | 0.750 | 1.29 (1.09-1.54) | 0.004 | 0.184 |
| ***Muscle power*** |  |  |  |  |  |  |  |
| Absolute power, W | 0.89 (0.74-1.06) | 0.200 | 1.01 (0.82-1.23) | 0.943 | 0.63 (0.46-0.86) | 0.004 | 0.021 |
| Allometric power, W/m² | 0.88 (0.74-1.05) | 0.148 | 0.99 (0.81-1.21) | 0.947 | 0.66 (0.49-0.90) | 0.008 | 0.050 |
| Relative power, W/kg | 0.85 (0.71-1.01) | 0.060 | 0.93 (0.76-1.14) | 0.468 | 0.69 (0.51-0.92) | 0.012 | 0.094 |
| ***Fat-to-muscle ratio (FMR)*** |  |  |  |  |  |  |  |
| Whole-body FMR | 1.00 (0.79-1.25) | 0.987 | 1.20 (0.99-1.45) | 0.067 | 0.81 (0.63-1.05) | 0.114 | 0.026 |
| Trunk FMR | 1.05 (0.87-1.28) | 0.599 | **1.26 (1.03-1.53)** | **0.024** | 0.82 (0.63-1.06) | 0.126 | 0.021 |
| Arm FMR | 0.91 (0.71-1.17) | 0.470 | 1.17 (0.98-1.41) | 0.089 | 0.77 (0.59-1.00) | 0.051 | 0.028 |
| Leg FMR | 0.93 (0.73-1.18) | 0.545 | 1.05 (0.87-1.27) | 0.609 | 0.89 (0.69-1.15) | 0.378 | 0.257 |
| ***Regional fat mass index (FMI)*** |  |  |  |  |  |  |  |
| Trunk FMI | 1.08 (0.90-1.28) | 0.418 | 1.25 (1.03-1.53) | 0.023 | 0.84 (0.64-1.10) | 0.201 | 0.053 |
| Arm FMI | 0.96 (0.78-1.19) | 0.706 | 1.15 (0.96-1.38) | 0.137 | 0.79 (0.60-1.03) | 0.081 | 0.075 |
| Leg FMI | 0.99 (0.80-1.22) | 0.919 | 1.09 (0.90-1.31) | 0.372 | 0.91 (0.70-1.18) | 0.479 | 0.232 |
| ***Regional-to-total fat ratio*** |  |  |  |  |  |  |  |
| Trunk-to-total fat ratio | 1.11 (0.94-1.32) | 0.213 | 1.32 (1.06-1.63) | 0.012 | 0.87 (0.68-1.12) | 0.279 | 0.092 |
| Arm-to-total fat ratio | 0.86 (0.71-1.03) | 0.104 | 0.96 (0.80-1.16) | 0.697 | 0.75 (0.58-0.96) | 0.024 | 0.416 |
| Leg-to-total fat ratio | 0.93 (0.78-1.11) | 0.418 | 0.80 (0.65-0.99) | 0.038 | 1.15 (0.89-1.47) | 0.287 | 0.165 |

Values were hazard ratios (95% CI) per 1-SD increase, estimated from Cox proportional-hazards models. Model 1 was adjusted for age in the sex-stratified analyses and additionally adjusted for sex in the total population. Model 2 was additionally adjusted for education level, smoking, alcohol drinking, physical activity, energy intake, diabetes, hypertension, cancer history and lipid-lowering medication use. Because of missing chair-stand data, analyses of chair stand time and muscle power included 3,370 participants (173 events; 1,674 men with 111 events and 1,696 women with 62 events); all other analyses used the full population shown. P values for interaction were derived from models containing the sex×exposure terms. Abbreviations: CHD, coronary heart disease; ALM, appendicular lean mass; BMI, body mass index; CI, confidence interval; FMI, fat mass index; FMR, fat-to-muscle ratio; HR, hazard ratio; PBF, percentage of body fat; SD, standard deviation; WC, waist circumference.

**Supplementary Table S13.** Sex-stratified associations of body composition, muscle strength, muscle power and regional fat distribution with incident stroke.

|  | **Total (n = 3,825)** | | **Male (n = 1,891)** | | **Female (n = 1,934)** | | **P for**  **interaction** |
| --- | --- | --- | --- | --- | --- | --- | --- |
| **Characteristic** | **HR (95% CI)** | **P** | **HR (95% CI)** | **P** | **HR (95% CI)** | **P** |  |
| No. of incident events | 150 | | 94 | | 56 | |  |
| **Model 1** |  |  |  |  |  |  |  |
| ***Body composition*** |  |  |  |  |  |  |  |
| PBF, % | 1.20 (0.95-1.51) | 0.120 | 1.22 (0.99-1.51) | 0.060 | 1.01 (0.78-1.31) | 0.928 | 0.271 |
| FMI, kg/m² | 1.21 (1.00-1.47) | 0.056 | 1.24 (1.02-1.52) | 0.035 | 1.08 (0.83-1.39) | 0.584 | 0.302 |
| ALM/weight, % | 0.79 (0.63-1.01) | 0.057 | 0.84 (0.68-1.03) | 0.092 | 0.90 (0.69-1.16) | 0.413 | 0.674 |
| ALM/height², kg/m² | 1.14 (0.93-1.39) | 0.204 | 1.14 (0.93-1.41) | 0.209 | 1.08 (0.83-1.40) | 0.559 | 0.960 |
| BMI, kg/m² | 1.20 (1.02-1.41) | 0.028 | 1.24 (1.01-1.52) | 0.042 | 1.14 (0.88-1.48) | 0.305 | 0.678 |
| WC, cm | 1.12 (0.96-1.32) | 0.156 | 1.20 (0.98-1.48) | 0.076 | 1.02 (0.79-1.32) | 0.857 | 0.320 |
| ***Muscle strength and function*** |  |  |  |  |  |  |  |
| Grip strength, kg | 0.89 (0.70-1.12) | 0.308 | 0.94 (0.76-1.17) | 0.574 | 0.88 (0.66-1.16) | 0.365 | 0.812 |
| Gait speed, m/s | 0.80 (0.67-0.95) | 0.012 | 0.82 (0.66-1.02) | 0.072 | 0.78 (0.60-1.03) | 0.078 | 0.974 |
| Five-times chair stand, s | 1.04 (0.89-1.21) | 0.626 | 1.08 (0.89-1.30) | 0.446 | 0.99 (0.77-1.28) | 0.940 | 0.460 |
| ***Muscle power*** |  |  |  |  |  |  |  |
| Absolute power, W | 0.97 (0.81-1.17) | 0.779 | 1.01 (0.81-1.26) | 0.913 | 0.91 (0.68-1.21) | 0.511 | 0.729 |
| Allometric power, W/m² | 0.97 (0.81-1.15) | 0.702 | 0.99 (0.80-1.23) | 0.943 | 0.93 (0.70-1.23) | 0.610 | 0.896 |
| Relative power, W/kg | 0.89 (0.74-1.06) | 0.191 | 0.91 (0.73-1.14) | 0.408 | 0.86 (0.65-1.14) | 0.293 | 0.827 |
| ***Fat-to-muscle ratio (FMR)*** |  |  |  |  |  |  |  |
| Whole-body FMR | 1.18 (0.93-1.48) | 0.167 | 1.21 (0.99-1.48) | 0.064 | 1.01 (0.78-1.31) | 0.913 | 0.208 |
| Trunk FMR | 1.13 (0.93-1.38) | 0.219 | 1.23 (1.00-1.51) | 0.047 | 0.95 (0.74-1.23) | 0.722 | 0.115 |
| Arm FMR | 1.14 (0.89-1.47) | 0.308 | 1.10 (0.91-1.33) | 0.327 | 1.07 (0.83-1.37) | 0.626 | 0.569 |
| Leg FMR | 1.18 (0.93-1.51) | 0.181 | 1.14 (0.94-1.39) | 0.176 | 1.08 (0.84-1.40) | 0.531 | 0.437 |
| ***Regional fat mass index (FMI)*** |  |  |  |  |  |  |  |
| Trunk FMI | 1.19 (0.99-1.42) | 0.059 | 1.27 (1.04-1.56) | 0.021 | 1.03 (0.79-1.34) | 0.819 | 0.203 |
| Arm FMI | 1.14 (0.93-1.41) | 0.214 | 1.13 (0.93-1.38) | 0.217 | 1.07 (0.83-1.39) | 0.596 | 0.565 |
| Leg FMI | 1.21 (0.98-1.49) | 0.078 | 1.18 (0.97-1.43) | 0.095 | 1.12 (0.87-1.44) | 0.383 | 0.467 |
| ***Regional-to-total fat ratio*** |  |  |  |  |  |  |  |
| Trunk-to-total fat ratio | 1.09 (0.92-1.29) | 0.335 | 1.22 (0.98-1.51) | 0.070 | 0.91 (0.71-1.17) | 0.467 | 0.098 |
| Arm-to-total fat ratio | 0.96 (0.79-1.17) | 0.681 | 0.88 (0.72-1.08) | 0.228 | 1.06 (0.82-1.37) | 0.678 | 0.212 |
| Leg-to-total fat ratio | 0.98 (0.82-1.17) | 0.810 | 0.90 (0.73-1.11) | 0.334 | 1.08 (0.84-1.40) | 0.529 | 0.280 |
| **Model 2** |  |  |  |  |  |  |  |
| ***Body composition*** |  |  |  |  |  |  |  |
| PBF, % | 1.18 (0.93-1.50) | 0.168 | 1.23 (0.99-1.54) | 0.059 | 0.96 (0.74-1.25) | 0.776 | 0.260 |
| FMI, kg/m² | 1.18 (0.97-1.45) | 0.104 | 1.25 (1.01-1.54) | 0.042 | 1.02 (0.78-1.32) | 0.911 | 0.311 |
| ALM/weight, % | 0.83 (0.65-1.06) | 0.131 | 0.85 (0.68-1.05) | 0.128 | 0.96 (0.73-1.25) | 0.738 | 0.698 |
| ALM/height², kg/m² | 1.12 (0.92-1.37) | 0.267 | 1.13 (0.91-1.39) | 0.268 | 1.08 (0.83-1.40) | 0.589 | 0.933 |
| BMI, kg/m² | 1.17 (0.99-1.38) | 0.068 | 1.22 (0.99-1.50) | 0.068 | 1.09 (0.84-1.41) | 0.529 | 0.695 |
| WC, cm | 1.09 (0.92-1.28) | 0.328 | 1.19 (0.96-1.47) | 0.105 | 0.95 (0.73-1.24) | 0.727 | 0.288 |
| ***Muscle strength and function*** |  |  |  |  |  |  |  |
| Grip strength, kg | 0.92 (0.73-1.16) | 0.493 | 0.96 (0.77-1.19) | 0.689 | 0.94 (0.71-1.26) | 0.695 | 0.796 |
| Gait speed, m/s | 0.84 (0.70-1.00) | 0.056 | 0.84 (0.67-1.04) | 0.113 | 0.90 (0.68-1.19) | 0.463 | 0.929 |
| Five-times chair stand, s | 1.01 (0.87-1.18) | 0.877 | 1.06 (0.88-1.28) | 0.525 | 0.89 (0.68-1.18) | 0.425 | 0.538 |
| ***Muscle power*** |  |  |  |  |  |  |  |
| Absolute power, W | 0.99 (0.82-1.19) | 0.898 | 1.00 (0.80-1.25) | 0.990 | 0.99 (0.74-1.33) | 0.959 | 0.681 |
| Allometric power, W/m² | 0.97 (0.82-1.17) | 0.780 | 0.98 (0.78-1.21) | 0.828 | 1.01 (0.76-1.35) | 0.930 | 0.865 |
| Relative power, W/kg | 0.91 (0.76-1.09) | 0.309 | 0.91 (0.73-1.13) | 0.390 | 0.96 (0.73-1.28) | 0.793 | 0.804 |
| ***Fat-to-muscle ratio (FMR)*** |  |  |  |  |  |  |  |
| Whole-body FMR | 1.16 (0.91-1.47) | 0.234 | 1.22 (0.99-1.50) | 0.064 | 0.96 (0.74-1.25) | 0.780 | 0.210 |
| Trunk FMR | 1.11 (0.90-1.37) | 0.314 | 1.24 (1.00-1.54) | 0.048 | 0.90 (0.70-1.18) | 0.452 | 0.102 |
| Arm FMR | 1.10 (0.85-1.43) | 0.461 | 1.09 (0.89-1.34) | 0.391 | 1.01 (0.78-1.31) | 0.961 | 0.633 |
| Leg FMR | 1.18 (0.92-1.51) | 0.184 | 1.16 (0.95-1.41) | 0.142 | 1.05 (0.82-1.36) | 0.692 | 0.472 |
| ***Regional fat mass index (FMI)*** |  |  |  |  |  |  |  |
| Trunk FMI | 1.16 (0.96-1.40) | 0.122 | 1.27 (1.03-1.58) | 0.027 | 0.97 (0.74-1.26) | 0.810 | 0.191 |
| Arm FMI | 1.10 (0.88-1.37) | 0.399 | 1.12 (0.91-1.37) | 0.295 | 1.00 (0.77-1.31) | 0.982 | 0.634 |
| Leg FMI | 1.20 (0.97-1.48) | 0.086 | 1.19 (0.98-1.45) | 0.081 | 1.08 (0.84-1.39) | 0.527 | 0.491 |
| ***Regional-to-total fat ratio*** |  |  |  |  |  |  |  |
| Trunk-to-total fat ratio | 1.05 (0.88-1.26) | 0.561 | 1.20 (0.96-1.50) | 0.113 | 0.87 (0.67-1.13) | 0.308 | 0.076 |
| Arm-to-total fat ratio | 0.92 (0.75-1.12) | 0.413 | 0.86 (0.70-1.06) | 0.154 | 1.00 (0.76-1.30) | 0.978 | 0.221 |
| Leg-to-total fat ratio | 1.03 (0.86-1.23) | 0.763 | 0.94 (0.76-1.17) | 0.583 | 1.14 (0.88-1.48) | 0.318 | 0.276 |

Values were hazard ratios (95% CI) per 1-SD increase, estimated from Cox proportional-hazards models. Model 1 was adjusted for age in the sex-stratified analyses and additionally adjusted for sex in the total population. Model 2 was additionally adjusted for education level, smoking, alcohol drinking, physical activity, energy intake, diabetes, hypertension, cancer history and lipid-lowering medication use. Because of missing chair-stand data, analyses of chair stand time and muscle power included 3,783 participants (148 events; 1,880 men with 92 events and 1,903 women with 56 events); all other analyses used the full population shown. P values for interaction were derived from models containing the sex×exposure terms. Abbreviations: ALM, appendicular lean mass; BMI, body mass index; CI, confidence interval; FMI, fat mass index; FMR, fat-to-muscle ratio; HR, hazard ratio; PBF, percentage of body fat; SD, standard deviation; WC, waist circumference.

**Supplementary Table S14.** Sex-stratified associations of body composition, muscle strength, muscle power and regional fat distribution with incident heart failure.

|  | **Total (n = 3,849)** | | **Male (n = 1,927)** | | **Female (n = 1,922)** | | **P for**  **interaction** |
| --- | --- | --- | --- | --- | --- | --- | --- |
| **Characteristic** | **HR (95% CI)** | **P** | **HR (95% CI)** | **P** | **HR (95% CI)** | **P** |  |
| No. of incident events | 130 | | 77 | | 53 | |  |
| **Model 1** |  |  |  |  |  |  |  |
| ***Body composition*** |  |  |  |  |  |  |  |
| PBF, % | 1.13 (0.89-1.44) | 0.306 | 1.32 (1.04-1.66) | 0.021 | 0.87 (0.68-1.11) | 0.246 | 0.020 |
| FMI, kg/m² | 1.22 (0.99-1.49) | 0.064 | 1.39 (1.11-1.73) | 0.004 | 0.97 (0.74-1.27) | 0.824 | 0.034 |
| ALM/weight, % | 0.82 (0.64-1.05) | 0.118 | 0.73 (0.58-0.92) | 0.008 | 1.11 (0.86-1.43) | 0.408 | 0.017 |
| ALM/height², kg/m² | 1.27 (1.03-1.56) | 0.025 | 1.23 (0.98-1.55) | 0.075 | 1.23 (0.95-1.59) | 0.118 | 0.698 |
| BMI, kg/m² | 1.27 (1.07-1.51) | 0.006 | 1.41 (1.13-1.77) | 0.002 | 1.11 (0.85-1.45) | 0.436 | 0.199 |
| WC, cm | 1.31 (1.11-1.56) | 0.002 | 1.38 (1.10-1.73) | 0.005 | 1.25 (0.97-1.62) | 0.088 | 0.527 |
| ***Muscle strength and function*** |  |  |  |  |  |  |  |
| Grip strength, kg | 0.73 (0.56-0.93) | 0.013 | 0.79 (0.62-1.01) | 0.056 | 0.81 (0.60-1.08) | 0.155 | 0.930 |
| Gait speed, m/s | 0.69 (0.57-0.83) | <0.001 | 0.78 (0.61-0.99) | 0.044 | 0.59 (0.45-0.78) | <0.001 | 0.258 |
| Five-times chair stand, s | 1.19 (1.05-1.36) | 0.007 | 1.12 (0.93-1.36) | 0.245 | 1.24 (1.05-1.47) | 0.012 | 0.700 |
| ***Muscle power*** |  |  |  |  |  |  |  |
| Absolute power, W | 0.86 (0.69-1.06) | 0.164 | 1.00 (0.79-1.29) | 0.969 | 0.63 (0.44-0.88) | 0.008 | 0.038 |
| Allometric power, W/m² | 0.86 (0.70-1.06) | 0.153 | 1.02 (0.80-1.30) | 0.876 | 0.64 (0.45-0.89) | 0.009 | 0.043 |
| Relative power, W/kg | 0.74 (0.60-0.91) | 0.005 | 0.86 (0.66-1.10) | 0.229 | 0.61 (0.44-0.83) | 0.002 | 0.089 |
| ***Fat-to-muscle ratio (FMR)*** |  |  |  |  |  |  |  |
| Whole-body FMR | 1.11 (0.87-1.41) | 0.393 | 1.31 (1.05-1.63) | 0.016 | 0.87 (0.67-1.13) | 0.300 | 0.016 |
| Trunk FMR | 1.10 (0.90-1.36) | 0.350 | 1.29 (1.03-1.62) | 0.026 | 0.89 (0.68-1.15) | 0.353 | 0.031 |
| Arm FMR | 1.18 (0.91-1.53) | 0.211 | 1.32 (1.08-1.61) | 0.006 | 0.96 (0.74-1.25) | 0.771 | 0.016 |
| Leg FMR | 1.01 (0.77-1.31) | 0.968 | 1.23 (1.00-1.52) | 0.054 | 0.83 (0.63-1.10) | 0.193 | 0.023 |
| ***Regional fat mass index (FMI)*** |  |  |  |  |  |  |  |
| Trunk FMI | 1.21 (1.01-1.47) | 0.044 | 1.38 (1.10-1.72) | 0.005 | 1.00 (0.76-1.30) | 0.981 | 0.068 |
| Arm FMI | 1.25 (1.00-1.55) | 0.048 | 1.37 (1.11-1.68) | 0.003 | 1.02 (0.78-1.34) | 0.863 | 0.033 |
| Leg FMI | 1.11 (0.89-1.39) | 0.362 | 1.28 (1.04-1.58) | 0.020 | 0.91 (0.69-1.20) | 0.503 | 0.035 |
| ***Regional-to-total fat ratio*** |  |  |  |  |  |  |  |
| Trunk-to-total fat ratio | 1.10 (0.91-1.32) | 0.311 | 1.13 (0.90-1.43) | 0.292 | 1.06 (0.82-1.38) | 0.654 | 0.750 |
| Arm-to-total fat ratio | 1.18 (0.96-1.45) | 0.122 | 1.14 (0.92-1.43) | 0.230 | 1.11 (0.85-1.44) | 0.435 | 0.780 |
| Leg-to-total fat ratio | 0.90 (0.74-1.08) | 0.266 | 0.91 (0.72-1.14) | 0.408 | 0.89 (0.69-1.17) | 0.415 | 0.993 |
| **Model 2** |  |  |  |  |  |  |  |
| ***Body composition*** |  |  |  |  |  |  |  |
| PBF, % | 0.97 (0.75-1.24) | 0.791 | 1.13 (0.88-1.44) | 0.341 | 0.82 (0.63-1.06) | 0.126 | 0.044 |
| FMI, kg/m² | 1.05 (0.85-1.31) | 0.643 | 1.18 (0.93-1.50) | 0.165 | 0.91 (0.69-1.21) | 0.536 | 0.089 |
| ALM/weight, % | 1.00 (0.77-1.30) | 0.990 | 0.87 (0.68-1.11) | 0.264 | 1.22 (0.94-1.60) | 0.140 | 0.033 |
| ALM/height², kg/m² | 1.19 (0.96-1.47) | 0.114 | 1.13 (0.89-1.43) | 0.303 | 1.23 (0.94-1.62) | 0.123 | 0.641 |
| BMI, kg/m² | 1.13 (0.94-1.35) | 0.189 | 1.21 (0.95-1.54) | 0.119 | 1.05 (0.80-1.40) | 0.711 | 0.307 |
| WC, cm | 1.17 (0.97-1.40) | 0.094 | 1.17 (0.92-1.49) | 0.208 | 1.20 (0.91-1.58) | 0.189 | 0.751 |
| ***Muscle strength and function*** |  |  |  |  |  |  |  |
| Grip strength, kg | 0.74 (0.57-0.96) | 0.023 | 0.80 (0.62-1.03) | 0.085 | 0.83 (0.61-1.11) | 0.210 | 0.990 |
| Gait speed, m/s | 0.73 (0.60-0.88) | 0.001 | 0.85 (0.65-1.10) | 0.209 | 0.59 (0.45-0.79) | <0.001 | 0.200 |
| Five-times chair stand, s | 1.17 (1.02-1.35) | 0.024 | 1.07 (0.88-1.30) | 0.501 | 1.26 (1.04-1.52) | 0.018 | 0.429 |
| ***Muscle power*** |  |  |  |  |  |  |  |
| Absolute power, W | 0.83 (0.66-1.03) | 0.096 | 0.98 (0.76-1.27) | 0.892 | 0.61 (0.43-0.87) | 0.006 | 0.028 |
| Allometric power, W/m² | 0.83 (0.68-1.02) | 0.083 | 0.99 (0.77-1.27) | 0.935 | 0.62 (0.44-0.88) | 0.007 | 0.035 |
| Relative power, W/kg | 0.77 (0.62-0.95) | 0.014 | 0.90 (0.70-1.17) | 0.442 | 0.61 (0.44-0.84) | 0.003 | 0.073 |
| ***Fat-to-muscle ratio (FMR)*** |  |  |  |  |  |  |  |
| Whole-body FMR | 0.95 (0.74-1.23) | 0.721 | 1.13 (0.89-1.43) | 0.302 | 0.83 (0.63-1.09) | 0.171 | 0.054 |
| Trunk FMR | 0.95 (0.76-1.18) | 0.617 | 1.09 (0.86-1.39) | 0.476 | 0.83 (0.63-1.09) | 0.181 | 0.074 |
| Arm FMR | 1.00 (0.76-1.31) | 0.999 | 1.15 (0.93-1.43) | 0.197 | 0.90 (0.68-1.18) | 0.439 | 0.081 |
| Leg FMR | 0.96 (0.73-1.26) | 0.767 | 1.16 (0.93-1.44) | 0.196 | 0.83 (0.63-1.10) | 0.188 | 0.065 |
| ***Regional fat mass index (FMI)*** |  |  |  |  |  |  |  |
| Trunk FMI | 1.04 (0.85-1.27) | 0.715 | 1.15 (0.90-1.47) | 0.265 | 0.92 (0.70-1.23) | 0.587 | 0.127 |
| Arm FMI | 1.07 (0.84-1.34) | 0.590 | 1.18 (0.94-1.47) | 0.145 | 0.95 (0.72-1.27) | 0.733 | 0.108 |
| Leg FMI | 1.05 (0.84-1.32) | 0.667 | 1.19 (0.95-1.48) | 0.123 | 0.91 (0.68-1.20) | 0.489 | 0.092 |
| ***Regional-to-total fat ratio*** |  |  |  |  |  |  |  |
| Trunk-to-total fat ratio | 0.94 (0.77-1.14) | 0.524 | 0.92 (0.71-1.18) | 0.493 | 0.98 (0.75-1.29) | 0.904 | 0.844 |
| Arm-to-total fat ratio | 1.07 (0.86-1.32) | 0.558 | 1.08 (0.86-1.35) | 0.508 | 1.02 (0.78-1.34) | 0.878 | 0.663 |
| Leg-to-total fat ratio | 1.05 (0.86-1.27) | 0.628 | 1.09 (0.86-1.39) | 0.464 | 0.98 (0.74-1.29) | 0.867 | 0.869 |

Values were hazard ratios (95% CI) per 1-SD increase, estimated from Cox proportional-hazards models. Model 1 was adjusted for age in the sex-stratified analyses and additionally adjusted for sex in the total population. Model 2 was additionally adjusted for education level, smoking, alcohol drinking, physical activity, energy intake, diabetes, hypertension, cancer history and lipid-lowering medication use. Because of missing chair-stand data, analyses of chair stand time and muscle power included 3,804 participants (127 events; 1,915 men with 76 events and 1,889 women with 51 events); all other analyses used the full population shown. P values for interaction were derived from models containing the sex×exposure terms. Abbreviations: ALM, appendicular lean mass; BMI, body mass index; CI, confidence interval; FMI, fat mass index; FMR, fat-to-muscle ratio; HR, hazard ratio; PBF, percentage of body fat; SD, standard deviation; WC, waist circumference.

**Supplementary Table S15a.** Sex-stratified associations with incident CVD after additional adjustment for measured blood pressure, serum creatinine and homocysteine.

|  | **Total (n = 2,406)** | | **Male (n = 1,129)** | | **Female (n = 1,277)** | | **P for**  **interaction** |
| --- | --- | --- | --- | --- | --- | --- | --- |
| **Characteristic** | **HR (95% CI)** | **P** | **HR (95% CI)** | **P** | **HR (95% CI)** | **P** |  |
| No. of incident events | 208 | | 120 | | 88 | |  |
| ***Body composition*** |  |  |  |  |  |  |  |
| PBF, % | 1.14 (0.92-1.40) | 0.226 | 1.26 (1.03-1.52) | 0.021 | 0.95 (0.77-1.18) | 0.642 | 0.022 |
| FMI, kg/m² | 1.15 (0.97-1.37) | 0.107 | 1.25 (1.05-1.50) | 0.014 | 1.02 (0.82-1.27) | 0.862 | 0.040 |
| ALM/weight, % | 0.83 (0.67-1.02) | 0.082 | 0.81 (0.67-0.99) | 0.037 | 0.95 (0.77-1.18) | 0.637 | 0.200 |
| ALM/height², kg/m² | 1.09 (0.91-1.30) | 0.336 | 1.11 (0.92-1.34) | 0.261 | 1.01 (0.81-1.26) | 0.907 | 0.414 |
| BMI, kg/m² | 1.14 (0.99-1.31) | 0.065 | 1.22 (1.02-1.46) | 0.033 | 1.06 (0.86-1.32) | 0.568 | 0.162 |
| WC, cm | 1.13 (0.99-1.31) | 0.079 | 1.20 (1.00-1.45) | 0.051 | 1.08 (0.87-1.33) | 0.503 | 0.237 |
| ***Muscle strength and function*** |  |  |  |  |  |  |  |
| Grip strength, kg | 0.76 (0.62-0.93) | 0.008 | 0.79 (0.65-0.96) | 0.016 | 0.88 (0.71-1.10) | 0.268 | 0.946 |
| Gait speed, m/s | 0.88 (0.75-1.02) | 0.090 | 0.85 (0.70-1.04) | 0.119 | 0.91 (0.73-1.14) | 0.421 | 0.670 |
| Five-times chair stand, s | 1.11 (0.98-1.25) | 0.115 | 1.00 (0.84-1.21) | 0.962 | 1.18 (1.01-1.39) | 0.043 | 0.286 |
| ***Muscle power*** |  |  |  |  |  |  |  |
| Absolute power, W | 0.93 (0.79-1.10) | 0.386 | 1.00 (0.82-1.22) | 0.977 | 0.81 (0.63-1.04) | 0.097 | 0.108 |
| Allometric power, W/m² | 0.94 (0.81-1.10) | 0.452 | 1.02 (0.84-1.24) | 0.835 | 0.82 (0.64-1.05) | 0.112 | 0.112 |
| Relative power, W/kg | 0.86 (0.73-1.02) | 0.076 | 0.92 (0.75-1.13) | 0.427 | 0.78 (0.61-0.99) | 0.042 | 0.222 |
| ***Fat-to-muscle ratio (FMR)*** |  |  |  |  |  |  |  |
| Whole-body FMR | 1.12 (0.91-1.38) | 0.283 | 1.24 (1.03-1.49) | 0.022 | 0.98 (0.79-1.22) | 0.849 | 0.025 |
| Trunk FMR | 1.13 (0.95-1.35) | 0.182 | 1.25 (1.04-1.52) | 0.020 | 1.00 (0.80-1.24) | 0.971 | 0.030 |
| Arm FMR | 1.14 (0.92-1.42) | 0.234 | 1.21 (1.01-1.44) | 0.035 | 1.06 (0.85-1.31) | 0.616 | 0.093 |
| Leg FMR | 1.02 (0.82-1.27) | 0.856 | 1.16 (0.97-1.38) | 0.107 | 0.94 (0.76-1.17) | 0.567 | 0.070 |
| ***Regional fat mass index (FMI)*** |  |  |  |  |  |  |  |
| Whole-body FMI | 1.15 (0.97-1.37) | 0.107 | 1.25 (1.05-1.50) | 0.014 | 1.02 (0.82-1.27) | 0.862 | 0.040 |
| Trunk FMI | 1.16 (0.99-1.36) | 0.060 | 1.27 (1.05-1.52) | 0.013 | 1.04 (0.84-1.29) | 0.731 | 0.057 |
| Arm FMI | 1.11 (0.93-1.33) | 0.256 | 1.19 (1.00-1.42) | 0.047 | 1.03 (0.83-1.28) | 0.808 | 0.107 |
| Leg FMI | 1.07 (0.90-1.29) | 0.442 | 1.19 (1.00-1.41) | 0.046 | 0.97 (0.78-1.21) | 0.799 | 0.046 |
| ***Regional-to-total fat ratio*** |  |  |  |  |  |  |  |
| Trunk-to-total fat ratio | 1.11 (0.95-1.29) | 0.204 | 1.16 (0.95-1.42) | 0.149 | 1.04 (0.84-1.28) | 0.743 | 0.330 |
| Arm-to-total fat ratio | 0.99 (0.84-1.18) | 0.930 | 0.98 (0.81-1.18) | 0.822 | 1.01 (0.82-1.25) | 0.919 | 0.548 |
| Leg-to-total fat ratio | 0.92 (0.79-1.07) | 0.288 | 0.92 (0.76-1.12) | 0.407 | 0.93 (0.75-1.15) | 0.481 | 0.829 |

Values were hazard ratios (95% CI) per 1-SD increase, estimated from Cox proportional-hazards models. Models were adjusted for age, sex, education level, smoking, alcohol drinking, physical activity, energy intake, diabetes, cancer history, lipid-lowering medication use, measured systolic and diastolic blood pressure, creatinine, and homocysteine. In the sex-stratified columns, sex was omitted because the analyses were stratified by it. Serum creatinine and homocysteine were available in 2,406 of the 3,168 participants (208 events). Owing to missing chair-stand data, analyses of five-times chair stand time and muscle power included 2,385 participants (204 events; 1,125 men with 118 events and 1,260 women with 86 events). P values for interaction were derived from models containing the sex × exposure term. Abbreviations: HR, hazard ratio; CI, confidence interval; CVD, cardiovascular disease; ALM, appendicular lean mass; BMI, body mass index; FMI, fat mass index; FMR, fat-to-muscle ratio; PBF, percentage of body fat; SD, standard deviation; WC, waist circumference.

**Supplementary Table S15b.** Sex-stratified associations with incident CHD after additional adjustment for measured blood pressure, serum creatinine and homocysteine.

|  | **Total (n = 2,585)** | | **Male (n = 1,217)** | | **Female (n = 1,368)** | | **P for**  **interaction** |
| --- | --- | --- | --- | --- | --- | --- | --- |
| **Characteristic** | **HR (95% CI)** | **P** | **HR (95% CI)** | **P** | **HR (95% CI)** | **P** |  |
| No. of incident events | 119 | | 70 | | 49 | |  |
| ***Body composition*** |  |  |  |  |  |  |  |
| PBF, % | 0.92 (0.71-1.21) | 0.565 | 1.12 (0.87-1.45) | 0.363 | 0.79 (0.60-1.04) | 0.090 | 0.063 |
| FMI, kg/m² | 0.98 (0.78-1.24) | 0.892 | 1.16 (0.91-1.47) | 0.241 | 0.83 (0.62-1.13) | 0.237 | 0.101 |
| ALM/weight, % | 1.14 (0.86-1.51) | 0.364 | 1.02 (0.79-1.30) | 0.896 | 1.17 (0.89-1.55) | 0.259 | 0.378 |
| ALM/height², kg/m² | 1.15 (0.91-1.45) | 0.241 | 1.25 (0.98-1.59) | 0.079 | 0.95 (0.70-1.27) | 0.710 | 0.276 |
| BMI, kg/m² | 1.03 (0.85-1.25) | 0.729 | 1.18 (0.92-1.51) | 0.185 | 0.87 (0.64-1.18) | 0.372 | 0.149 |
| WC, cm | 1.03 (0.85-1.24) | 0.771 | 1.14 (0.89-1.46) | 0.292 | 0.92 (0.68-1.23) | 0.556 | 0.285 |
| ***Muscle strength and function*** |  |  |  |  |  |  |  |
| Grip strength, kg | 0.89 (0.68-1.17) | 0.396 | 0.90 (0.70-1.16) | 0.427 | 0.94 (0.69-1.27) | 0.676 | 0.846 |
| Gait speed, m/s | 0.91 (0.74-1.11) | 0.337 | 0.92 (0.71-1.20) | 0.551 | 0.88 (0.65-1.19) | 0.419 | 0.799 |
| Five-times chair stand, s | 1.18 (1.02-1.38) | 0.030 | 1.03 (0.81-1.31) | 0.835 | 1.28 (1.07-1.55) | 0.009 | 0.237 |
| ***Muscle power*** |  |  |  |  |  |  |  |
| Absolute power, W | 0.88 (0.70-1.11) | 0.284 | 1.04 (0.80-1.34) | 0.792 | 0.60 (0.42-0.88) | 0.008 | 0.011 |
| Allometric power, W/m² | 0.87 (0.70-1.07) | 0.191 | 1.03 (0.79-1.33) | 0.838 | 0.61 (0.43-0.88) | 0.008 | 0.017 |
| Relative power, W/kg | 0.83 (0.67-1.04) | 0.103 | 0.95 (0.73-1.24) | 0.689 | 0.66 (0.47-0.93) | 0.016 | 0.066 |
| ***Fat-to-muscle ratio (FMR)*** |  |  |  |  |  |  |  |
| Whole-body FMR | 0.90 (0.68-1.19) | 0.468 | 1.10 (0.86-1.41) | 0.432 | 0.81 (0.60-1.08) | 0.147 | 0.115 |
| Trunk FMR | 1.00 (0.79-1.27) | 0.967 | 1.18 (0.92-1.52) | 0.192 | 0.86 (0.64-1.15) | 0.308 | 0.104 |
| Arm FMR | 0.82 (0.61-1.11) | 0.198 | 1.10 (0.87-1.39) | 0.442 | 0.75 (0.56-1.01) | 0.060 | 0.133 |
| Leg FMR | 0.80 (0.59-1.08) | 0.151 | 0.95 (0.74-1.21) | 0.666 | 0.82 (0.61-1.11) | 0.203 | 0.585 |
| ***Regional fat mass index (FMI)*** |  |  |  |  |  |  |  |
| Whole-body FMI | 0.98 (0.78-1.24) | 0.892 | 1.16 (0.91-1.47) | 0.241 | 0.83 (0.62-1.13) | 0.237 | 0.101 |
| Trunk FMI | 1.04 (0.84-1.29) | 0.733 | 1.20 (0.94-1.54) | 0.146 | 0.87 (0.64-1.17) | 0.354 | 0.115 |
| Arm FMI | 0.89 (0.69-1.16) | 0.394 | 1.12 (0.89-1.42) | 0.329 | 0.76 (0.55-1.03) | 0.075 | 0.104 |
| Leg FMI | 0.91 (0.70-1.17) | 0.455 | 1.03 (0.82-1.31) | 0.782 | 0.85 (0.63-1.16) | 0.306 | 0.306 |
| ***Regional-to-total fat ratio*** |  |  |  |  |  |  |  |
| Trunk-to-total fat ratio | 1.13 (0.92-1.39) | 0.256 | 1.28 (0.98-1.68) | 0.074 | 0.97 (0.73-1.29) | 0.842 | 0.305 |
| Arm-to-total fat ratio | 0.83 (0.66-1.04) | 0.109 | 1.00 (0.78-1.28) | 0.991 | 0.71 (0.53-0.94) | 0.017 | 0.285 |
| Leg-to-total fat ratio | 0.90 (0.73-1.11) | 0.320 | 0.79 (0.61-1.03) | 0.084 | 1.04 (0.78-1.37) | 0.803 | 0.336 |

Values were hazard ratios (95% CI) per 1-SD increase, estimated from Cox proportional-hazards models. Models were adjusted for age, sex, education level, smoking, alcohol drinking, physical activity, energy intake, diabetes, cancer history, lipid-lowering medication use, measured systolic and diastolic blood pressure, creatinine, and homocysteine. In the sex-stratified columns, sex was omitted because the analyses were stratified by it. Serum creatinine and homocysteine were available in 2,585 of the 3,408 participants (119 events). Owing to missing chair-stand data, analyses of five-times chair stand time and muscle power included 2,560 participants (115 events; 1,211 men with 68 events and 1,349 women with 47 events). P values for interaction were derived from models containing the sex × exposure term. Abbreviations: HR, hazard ratio; CI, confidence interval; CHD, coronary heart disease; ALM, appendicular lean mass; BMI, body mass index; FMI, fat mass index; FMR, fat-to-muscle ratio; PBF, percentage of body fat; SD, standard deviation; WC, waist circumference.

**Supplementary Table S15c.** Sex-stratified associations with incident stroke after additional adjustment for measured blood pressure, serum creatinine and homocysteine.

|  | **Total (n = 2,880)** | | **Male (n = 1,361)** | | **Female (n = 1,519)** | | **P for**  **interaction** |
| --- | --- | --- | --- | --- | --- | --- | --- |
| **Characteristic** | **HR (95% CI)** | **P** | **HR (95% CI)** | **P** | **HR (95% CI)** | **P** |  |
| No. of incident events | 75 | | 47 | | 28 | |  |
| ***Body composition*** |  |  |  |  |  |  |  |
| PBF, % | 1.17 (0.84-1.63) | 0.361 | 1.21 (0.89-1.63) | 0.220 | 0.97 (0.68-1.38) | 0.867 | 0.266 |
| FMI, kg/m² | 1.17 (0.88-1.55) | 0.289 | 1.23 (0.92-1.65) | 0.157 | 1.00 (0.70-1.42) | 0.981 | 0.244 |
| ALM/weight, % | 0.82 (0.58-1.17) | 0.279 | 0.86 (0.64-1.16) | 0.314 | 0.95 (0.65-1.38) | 0.779 | 0.679 |
| ALM/height², kg/m² | 1.12 (0.84-1.50) | 0.426 | 1.14 (0.85-1.53) | 0.387 | 1.05 (0.73-1.52) | 0.777 | 0.623 |
| BMI, kg/m² | 1.16 (0.92-1.46) | 0.205 | 1.22 (0.91-1.63) | 0.181 | 1.06 (0.75-1.50) | 0.736 | 0.449 |
| WC, cm | 1.09 (0.86-1.37) | 0.477 | 1.24 (0.93-1.66) | 0.142 | 0.88 (0.61-1.26) | 0.478 | 0.104 |
| ***Muscle strength and function*** |  |  |  |  |  |  |  |
| Grip strength, kg | 0.89 (0.64-1.25) | 0.509 | 0.97 (0.72-1.32) | 0.859 | 0.88 (0.59-1.31) | 0.519 | 0.468 |
| Gait speed, m/s | 0.71 (0.55-0.92) | 0.008 | 0.65 (0.47-0.89) | 0.008 | 0.87 (0.58-1.30) | 0.500 | 0.391 |
| Five-times chair stand, s | 1.03 (0.82-1.29) | 0.811 | 1.08 (0.82-1.41) | 0.591 | 0.94 (0.64-1.38) | 0.747 | 0.633 |
| ***Muscle power*** |  |  |  |  |  |  |  |
| Absolute power, W | 1.00 (0.78-1.30) | 0.981 | 1.00 (0.74-1.36) | 0.980 | 1.04 (0.70-1.55) | 0.837 | 0.931 |
| Allometric power, W/m² | 0.97 (0.76-1.24) | 0.799 | 0.95 (0.70-1.29) | 0.740 | 1.05 (0.71-1.54) | 0.818 | 0.884 |
| Relative power, W/kg | 0.90 (0.69-1.16) | 0.400 | 0.86 (0.62-1.18) | 0.348 | 1.02 (0.69-1.49) | 0.938 | 0.651 |
| ***Fat-to-muscle ratio (FMR)*** |  |  |  |  |  |  |  |
| Whole-body FMR | 1.13 (0.80-1.59) | 0.480 | 1.19 (0.89-1.59) | 0.247 | 0.96 (0.67-1.39) | 0.846 | 0.244 |
| Trunk FMR | 1.06 (0.80-1.42) | 0.673 | 1.21 (0.89-1.63) | 0.220 | 0.88 (0.61-1.27) | 0.494 | 0.101 |
| Arm FMR | 1.10 (0.75-1.60) | 0.630 | 1.06 (0.80-1.42) | 0.682 | 1.06 (0.74-1.52) | 0.759 | 0.755 |
| Leg FMR | 1.23 (0.87-1.76) | 0.246 | 1.15 (0.87-1.51) | 0.314 | 1.10 (0.77-1.57) | 0.603 | 0.591 |
| ***Regional fat mass index (FMI)*** |  |  |  |  |  |  |  |
| Whole-body FMI | 1.17 (0.88-1.55) | 0.289 | 1.23 (0.92-1.65) | 0.157 | 1.00 (0.70-1.42) | 0.981 | 0.244 |
| Trunk FMI | 1.12 (0.86-1.45) | 0.404 | 1.25 (0.93-1.68) | 0.144 | 0.92 (0.64-1.32) | 0.653 | 0.127 |
| Arm FMI | 1.05 (0.77-1.44) | 0.739 | 1.08 (0.81-1.45) | 0.609 | 1.00 (0.70-1.43) | 0.991 | 0.647 |
| Leg FMI | 1.25 (0.94-1.68) | 0.130 | 1.22 (0.93-1.60) | 0.145 | 1.12 (0.79-1.57) | 0.530 | 0.414 |
| ***Regional-to-total fat ratio*** |  |  |  |  |  |  |  |
| Trunk-to-total fat ratio | 0.98 (0.76-1.26) | 0.866 | 1.15 (0.83-1.58) | 0.395 | 0.80 (0.57-1.15) | 0.227 | 0.069 |
| Arm-to-total fat ratio | 0.85 (0.63-1.13) | 0.258 | 0.79 (0.58-1.07) | 0.130 | 0.99 (0.69-1.44) | 0.978 | 0.212 |
| Leg-to-total fat ratio | 1.14 (0.89-1.47) | 0.293 | 1.02 (0.75-1.38) | 0.909 | 1.24 (0.88-1.75) | 0.226 | 0.339 |

Values were hazard ratios (95% CI) per 1-SD increase, estimated from Cox proportional-hazards models. Models were adjusted for age, sex, education level, smoking, alcohol drinking, physical activity, energy intake, diabetes, cancer history, lipid-lowering medication use, measured systolic and diastolic blood pressure, creatinine, and homocysteine. In the sex-stratified columns, sex was omitted because the analyses were stratified by it. Serum creatinine and homocysteine were available in 2,880 of the 3,825 participants (75 events). Owing to missing chair-stand data, analyses of five-times chair stand time and muscle power included 2,856 participants (75 events; 1,356 men with 47 events and 1,500 women with 28 events). P values for interaction were derived from models containing the sex × exposure term. Abbreviations: HR, hazard ratio; CI, confidence interval; ALM, appendicular lean mass; BMI, body mass index; FMI, fat mass index; FMR, fat-to-muscle ratio; PBF, percentage of body fat; SD, standard deviation; WC, waist circumference.

**Supplementary Table S15d.** Sex-stratified associations with incident heart failure after additional adjustment for measured blood pressure, serum creatinine and homocysteine.

|  | **Total (n = 2,882)** | | **Male (n = 1,374)** | | **Female (n = 1,508)** | | **P for**  **interaction** |
| --- | --- | --- | --- | --- | --- | --- | --- |
| **Characteristic** | **HR (95% CI)** | **P** | **HR (95% CI)** | **P** | **HR (95% CI)** | **P** |  |
| No. of incident events | 77 | | 42 | | 35 | |  |
| ***Body composition*** |  |  |  |  |  |  |  |
| PBF, % | 1.20 (0.86-1.68) | 0.285 | 1.23 (0.88-1.72) | 0.234 | 1.05 (0.76-1.46) | 0.754 | 0.380 |
| FMI, kg/m² | 1.32 (1.00-1.73) | 0.049 | 1.36 (0.99-1.86) | 0.056 | 1.19 (0.85-1.66) | 0.302 | 0.336 |
| ALM/weight, % | 0.85 (0.60-1.21) | 0.374 | 0.87 (0.62-1.23) | 0.437 | 0.92 (0.66-1.28) | 0.626 | 0.634 |
| ALM/height², kg/m² | 1.49 (1.13-1.97) | 0.005 | 1.44 (1.06-1.97) | 0.021 | 1.36 (0.97-1.90) | 0.077 | 0.923 |
| BMI, kg/m² | 1.40 (1.12-1.76) | 0.004 | 1.48 (1.08-2.03) | 0.015 | 1.37 (0.97-1.92) | 0.070 | 0.582 |
| WC, cm | 1.44 (1.14-1.82) | 0.002 | 1.44 (1.04-2.00) | 0.026 | 1.55 (1.09-2.19) | 0.013 | 0.905 |
| ***Muscle strength and function*** |  |  |  |  |  |  |  |
| Grip strength, kg | 0.84 (0.59-1.19) | 0.319 | 0.86 (0.61-1.22) | 0.402 | 0.96 (0.66-1.38) | 0.809 | 0.483 |
| Gait speed, m/s | 0.68 (0.53-0.87) | 0.002 | 0.85 (0.60-1.19) | 0.338 | 0.52 (0.37-0.73) | <0.001 | 0.177 |
| Five-times chair stand, s | 1.16 (0.98-1.38) | 0.088 | 0.92 (0.67-1.27) | 0.624 | 1.30 (1.05-1.60) | 0.015 | 0.167 |
| ***Muscle power*** |  |  |  |  |  |  |  |
| Absolute power, W | 1.05 (0.79-1.38) | 0.753 | 1.31 (0.95-1.82) | 0.100 | 0.66 (0.43-1.01) | 0.055 | 0.041 |
| Allometric power, W/m² | 1.04 (0.80-1.34) | 0.776 | 1.32 (0.97-1.81) | 0.082 | 0.68 (0.45-1.03) | 0.066 | 0.035 |
| Relative power, W/kg | 0.86 (0.65-1.13) | 0.268 | 1.13 (0.81-1.58) | 0.469 | 0.55 (0.36-0.83) | 0.004 | 0.020 |
| ***Fat-to-muscle ratio (FMR)*** |  |  |  |  |  |  |  |
| Whole-body FMR | 1.18 (0.85-1.63) | 0.314 | 1.21 (0.88-1.66) | 0.235 | 1.07 (0.77-1.48) | 0.698 | 0.345 |
| Trunk FMR | 1.14 (0.86-1.51) | 0.357 | 1.18 (0.85-1.64) | 0.320 | 1.08 (0.77-1.51) | 0.649 | 0.452 |
| Arm FMR | 1.28 (0.92-1.79) | 0.138 | 1.27 (0.95-1.70) | 0.113 | 1.17 (0.85-1.60) | 0.336 | 0.249 |
| Leg FMR | 1.08 (0.77-1.51) | 0.665 | 1.18 (0.87-1.59) | 0.282 | 0.96 (0.69-1.34) | 0.829 | 0.265 |
| ***Regional fat mass index (FMI)*** |  |  |  |  |  |  |  |
| Whole-body FMI | 1.32 (1.00-1.73) | 0.049 | 1.36 (0.99-1.86) | 0.056 | 1.19 (0.85-1.66) | 0.302 | 0.336 |
| Trunk FMI | 1.29 (1.00-1.66) | 0.053 | 1.31 (0.94-1.83) | 0.104 | 1.25 (0.89-1.76) | 0.196 | 0.581 |
| Arm FMI | 1.39 (1.05-1.84) | 0.023 | 1.37 (1.03-1.84) | 0.032 | 1.27 (0.91-1.76) | 0.157 | 0.265 |
| Leg FMI | 1.20 (0.90-1.59) | 0.206 | 1.31 (0.99-1.73) | 0.062 | 1.03 (0.74-1.44) | 0.853 | 0.149 |
| ***Regional-to-total fat ratio*** |  |  |  |  |  |  |  |
| Trunk-to-total fat ratio | 1.03 (0.79-1.33) | 0.847 | 0.92 (0.65-1.29) | 0.621 | 1.20 (0.84-1.71) | 0.322 | 0.532 |
| Arm-to-total fat ratio | 1.31 (0.99-1.73) | 0.056 | 1.23 (0.89-1.71) | 0.202 | 1.27 (0.91-1.76) | 0.158 | 0.954 |
| Leg-to-total fat ratio | 0.95 (0.74-1.23) | 0.706 | 1.09 (0.79-1.51) | 0.597 | 0.80 (0.56-1.15) | 0.234 | 0.395 |

Values were hazard ratios (95% CI) per 1-SD increase, estimated from Cox proportional-hazards models. Models were adjusted for age, sex, education level, smoking, alcohol drinking, physical activity, energy intake, diabetes, cancer history, lipid-lowering medication use, measured systolic and diastolic blood pressure, creatinine, and homocysteine. In the sex-stratified columns, sex was omitted because the analyses were stratified by it. Serum creatinine and homocysteine were available in 2,882 of the 3,849 participants (77 events). Owing to missing chair-stand data, analyses of five-times chair stand time and muscle power included 2,856 participants (77 events; 1,369 men with 42 events and 1,487 women with 35 events). P values for interaction were derived from models containing the sex × exposure term. Abbreviations: HR, hazard ratio; CI, confidence interval; CHD, coronary heart disease; ALM, appendicular lean mass; BMI, body mass index; FMI, fat mass index; FMR, fat-to-muscle ratio; PBF, percentage of body fat; SD, standard deviation; WC, waist circumference.

**Supplementary Table S16. Mutually adjusted models for ratio-based body composition measures, adjusted for standardized adiposity or muscle indices.**

|  | **Male (n = 1548)** | | **Female (n = 1620)** | |
| --- | --- | --- | --- | --- |
| **Characteristic** | **HR (95% CI)** | **P** | **HR (95% CI)** | **P** |
| No. of incident CVD | 199 | | 131 | |
| **ALM/weight** |  |  |  |  |
| Single-exposure (reference) | **0.80 (0.69-0.93)** | **0.004** | 1.06 (0.89-1.27) | 0.483 |
| + FMI (mutually adjusted) | 0.89 (0.70-1.14) | 0.357 | 1.05 (0.80-1.37) | 0.728 |
| **Whole-body FMR** |  |  |  |  |
| Single-exposure (reference) | **1.22 (1.06-1.42)** | **0.006** | 0.91 (0.77-1.09) | 0.301 |
| + ALMI (mutually adjusted) | **1.21 (1.05-1.41)** | **0.011** | 0.91 (0.76-1.08) | 0.282 |
| **Trunk FMR** |  |  |  |  |
| Single-exposure (reference) | **1.25 (1.07-1.45)** | **0.004** | 0.90 (0.75-1.07) | 0.232 |
| + ALMI (mutually adjusted) | **1.24 (1.06-1.45)** | **0.007** | 0.89 (0.75-1.07) | 0.210 |
| **Arm FMR** |  |  |  |  |
| Single-exposure (reference) | **1.19 (1.03-1.36)** | **0.014** | 0.98 (0.82-1.16) | 0.781 |
| + ALMI (mutually adjusted) | **1.18 (1.03-1.35)** | **0.021** | 0.97 (0.82-1.17) | 0.774 |
| **Leg FMR** |  |  |  |  |
| Single-exposure (reference) | 1.12 (0.97-1.28) | 0.118 | 0.94 (0.79-1.12) | 0.494 |
| + ALMI (mutually adjusted) | 1.11 (0.97-1.28) | 0.132 | 0.94 (0.79-1.12) | 0.492 |
| **Trunk-to-total fat ratio** |  |  |  |  |
| Single-exposure (reference) | **1.23 (1.05-1.45)** | **0.009** | 0.92 (0.77-1.09) | 0.320 |
| + ALMI (mutually adjusted) | **1.22 (1.04-1.44)** | **0.016** | 0.91 (0.77-1.08) | 0.304 |
| **Arm-to-total fat ratio** |  |  |  |  |
| Single-exposure (reference) | 0.96 (0.83-1.10) | 0.553 | 0.98 (0.83-1.17) | 0.846 |
| + ALMI (mutually adjusted) | 0.95 (0.82-1.09) | 0.444 | 0.98 (0.82-1.17) | 0.847 |
| **Leg-to-total fat ratio** |  |  |  |  |
| Single-exposure (reference) | 0.87 (0.75-1.01) | 0.070 | 1.05 (0.88-1.25) | 0.623 |
| + ALMI (mutually adjusted) | 0.88 (0.75-1.02) | 0.097 | 1.05 (0.88-1.25) | 0.611 |

Values were hazard ratios (95% CI) per 1-SD increase from Cox proportional-hazards models. All models were adjusted for age, education level, smoking, alcohol drinking, physical activity, energy intake, diabetes, hypertension, cancer history and lipid-lowering medication use. Abbreviations: FMI, fat mass index; ALM, appendicular lean mass; ALMI, appendicular lean mass index (ALM/height²); FMR, fat-to-muscle ratio. Mutually adjusted models for ALM/weight included FMI as the adjustment variable; mutually adjusted models for FMR and fat ratios included ALMI as the adjustment variable.

**Supplementary Table S17. Sex-stratified mutually adjusted associations of STS-derived muscle power with CVD risk, additionally adjusted for adiposity (FMI) and muscle mass (ALM/height²).**

|  | **Male (n = 1542)** | | **Female (n = 1593)** | |
| --- | --- | --- | --- | --- |
| **Characteristic** | **HR (95% CI)** | **P** | **HR (95% CI)** | **P** |
| No. of incident CVD | 196 | | 129 | |
| Absolute power (W) |  |  |  |  |
| Single-exposure (**reference, from Table 2 Model 2**) | 0.99 (0.85-1.15) | 0.906 | 0.77 (0.62-0.95) | 0.013 |
| + FMI **+ ALM/height²** (simultaneously adjusted) | 0.92 (0.78-1.09) | 0.336 | 0.75 (0.61-0.94) | 0.012 |
| Allometric power (W/m²) |  |  |  |  |
| Single-exposure (**reference, from Table 2 Model 2**) | 1.00 (0.86-1.16) | 0.988 | 0.79 (0.64-0.96) | 0.021 |
| + FMI **+ ALM/height²** (simultaneously adjusted) | 0.93 (0.79-1.09) | 0.352 | 0.77 (0.62-0.96) | 0.019 |
| Relative power (W/kg) |  |  |  |  |
| Single-exposure (**reference, from Table 2 Model 2**) | 0.92 (0.79-1.07) | 0.297 | 0.77 (0.63-0.94) | 0.011 |
| + FMI **+ ALM/height²** (simultaneously adjusted) | 0.93 (0.80-1.09) | 0.371 | 0.77 (0.63-0.93) | 0.008 |

Values were hazard ratios (95% CI) per 1-SD increase from Cox proportional-hazards models. All models were adjusted for age, education level, smoking, alcohol drinking, physical activity, energy intake, diabetes, hypertension, cancer history and lipid-lowering medication use. Abbreviations: FMI, fat mass index; ALM, appendicular lean mass.

**Supplementary Table S18.** Sex-stratified associations of STS-derived muscle power with CVD risk after excluding cardiovascular events within the first two years of follow-up.

|  | **Male (n = 1456)** | | **Female (n = 1549)** | |
| --- | --- | --- | --- | --- |
| **Characteristic** | **HR (95% CI)** | **P** | **HR (95% CI)** | **P** |
| No. of incident CVD | 131 | | 102 | |
| Absolute power (W) |  |  |  |  |
| Model 2 (single-exposure) | 0.99 (0.82-1.20) | 0.957 | 0.79 (0.62-0.99) | 0.045 |
| + FMI + ALM/height² (simultaneously adjusted) | 0.94 (0.76-1.15) | 0.521 | 0.76 (0.60-0.97) | 0.028 |
| Allometric power (W/m²) |  |  |  |  |
| Model 2 (single-exposure) | 1.01 (0.84-1.21) | 0.921 | 0.79 (0.63-1.00) | 0.051 |
| + FMI + ALM/height² (simultaneously adjusted) | 0.94 (0.77-1.16) | 0.584 | 0.76 (0.60-0.97) | 0.030 |
| Relative power (W/kg) |  |  |  |  |
| Model 2 (single-exposure) | 0.94 (0.78-1.14) | 0.535 | 0.76 (0.61-0.95) | 0.018 |
| + FMI + ALM/height² (simultaneously adjusted) | 0.95 (0.79-1.15) | 0.598 | 0.76 (0.61-0.96) | 0.019 |

Values were hazard ratios (95% CI) per 1-SD increase from Cox proportional-hazards models. After exclusions, the analysis included 3,005 participants (1,456 men with 131 events and 1,549 women with 102 events). All models were adjusted for age, education level, smoking, alcohol drinking, physical activity, energy intake, diabetes, hypertension, cancer history and lipid-lowering medication use. Abbreviations: FMI, fat mass index; ALM, appendicular lean mass; STS, sit-to-stand.
